# Artificial intelligence-enabled ECG for left ventricular diastolic function and filling pressure

**DOI:** 10.1038/s41746-023-00993-7

**Published:** 2024-01-06

**Authors:** Eunjung Lee, Saki Ito, William R. Miranda, Francisco Lopez-Jimenez, Garvan C. Kane, Samuel J. Asirvatham, Peter A. Noseworthy, Paul A. Friedman, Rickey E. Carter, Barry A. Borlaug, Zachi I. Attia, Jae K. Oh

**Affiliations:** 1 Department of Cardiovascular Medicine, Mayo Clinic, Rochester, MN USA; 2 Health Sciences Research, Mayo Clinic, Jacksonville, FL USA

**Keywords:** Cardiovascular diseases, Outcomes research

## Abstract

Assessment of left ventricular diastolic function plays a major role in the diagnosis and prognosis of cardiac diseases, including heart failure with preserved ejection fraction. We aimed to develop an artificial intelligence (AI)-enabled electrocardiogram (ECG) model to identify echocardiographically determined diastolic dysfunction and increased filling pressure. We trained, validated, and tested an AI-enabled ECG in 98,736, 21,963, and 98,763 patients, respectively, who had an ECG and echocardiographic diastolic function assessment within 14 days with no exclusion criteria. It was also tested in 55,248 patients with indeterminate diastolic function by echocardiography. The model was evaluated using the area under the curve (AUC) of the receiver operating characteristic curve, and its prognostic performance was compared to echocardiography. The AUC for detecting increased filling pressure was 0.911. The AUCs to identify diastolic dysfunction grades ≥1, ≥2, and 3 were 0.847, 0.911, and 0.943, respectively. During a median follow-up of 5.9 years, 20,223 (20.5%) died. Patients with increased filling pressure predicted by AI-ECG had higher mortality than those with normal filling pressure, after adjusting for age, sex, and comorbidities in the test group (hazard ratio (HR) 1.7, 95% CI 1.645–1.757) similar to echocardiography and in the indeterminate group (HR 1.34, 95% CI 1.298–1.383). An AI-enabled ECG identifies increased filling pressure and diastolic function grades with a good prognostic value similar to echocardiography. AI-ECG is a simple and promising tool to enhance the detection of diseases associated with diastolic dysfunction and increased diastolic filling pressure.

## Introduction

Left ventricular diastolic function becomes abnormal in myocardial diseases, and with the worsening of diastolic function, filling pressure rises even in the setting of preserved ejection fraction^1^. Documentation of increased left ventricular filling pressure is, therefore, a key component in the diagnosis of heart failure with preserved ejection fraction which is becoming a major cardiovascular condition^1–3^. Moreover, elevated diastolic filling pressure has been associated with heart failure symptoms and higher mortality in patients with myocardial infarction, valvular diseases, and cardiomyopathies^4–7^.

Diastolic function is most commonly evaluated by echocardiography^8^. However, assessment of diastolic function requires a skilled sonographer and a cardiologist with advanced training in echocardiographic interpretation, and results of diastolic function assessment are equivocal in a substantial proportion of patients^9^.

Twelve-lead electrocardiography (ECG) is widely performed with little cost and recent studies have shown that artificial intelligence (AI)-enabled ECG accurately detects various cardiac disorders that have historically required more advanced imaging to detect^10–12^. We sought to develop and validate a deep neural network based on 12-lead ECG to predict diastolic function and increased filling pressure determined by echocardiography, and then evaluate its associations with all-cause mortality.

## Results

For this study, 274,710 patients who had an ECG and echocardiographic diastolic function assessment within 14 days were identified with no exclusion criteria. Using the recommended algorithm, echocardiography determination of diastolic function was possible in 219,462 patients (80%) but was indeterminate in 55,248 patients (20%). Baseline patient characteristics were similar among training, validation, and testing groups (Supplemental Table 1). There were 20,264 patients with left ventricular ejection fraction <50% (9.2%), 15,548 patients with cardiac amyloidosis (7.1%), 8,161 patients with hypertrophic cardiomyopathy (3.7%), and 5409 patients with moderate to severe aortic stenosis (2.5%). In the test set, estimated diastolic filling pressure by echocardiography was normal in 76,880 patients including 57,301 (58%) with normal diastolic function and 19,579 (19.8%) grade 1 dysfunction, and increased in 21,883 patients including 17,815 (18%) grade 2 and 4068 (4.1%) grade 3 dysfunction. Patients with increased filling pressure determined by echocardiography were older and had more comorbidities (*p* < 0.001, Table 1). Similarly, patients identified as having increased filling pressure by AI-ECG had more comorbidities (*p* < 0.001, Supplemental Table 2).

**Table 1:** Patient characteristics of four diastolic grade groups determined by echocardiography in test set.

	Test (*n* = 98,763)			
	Normal (*n* = 57,301)	Grade 1 (*n* = 19,579)	Grade 2 (*n* = 17,815)	Grade 3 (*n* = 4068)
Age, year	54.0 ± 16.0	69.0 ± 10.8	73.9 ± 12.4	69.2 ± 14.6
Female sex, number (%)	28,873 (50.4%)	9678 (49.4%)	10,049 (56.4%)	1493 (36.7%)
Myocardial infarction, number (%)	3269 (5.7%)	1735 (8.9%)	2746 (15.5%)	705 (17.4%)
Congestive heart failure, number (%)	4774 (8.4%)	2598 (13.3%)	6777 (38.1%)	2633 (64.9%)
Cerebrovascular disease, number (%)	3835 (6.7%)	2340 (12.0%)	2537 (14.3%)	465 (11.5%)
Chronic pulmonary disease, number (%)	6444 (11.3%)	3097 (15.9%)	4370 (24.6%)	1051 (25.9%)
Diabetes, number (%)	5778 (10.1%)	3356 (17.1%)	5086 (28.5%)	1206 (29.6%)
Renal disease, number (%)	4786 (8.4%)	2633 (13.5%)	5288 (29.8%)	1406 (34.7%)
Hypertension, number (%)	19,673 (34.4%)	10,779 (55.2%)	11,990 (67.5%)	2378 (58.6%)
Obesity, number (%)	18,141 (31.7%)	6630 (33.9%)	6385 (35.8%)	1423 (35.0%)

Values are *n* (%) or mean ± SD. Obesity was defined as body mass index ≥30. Renal disease includes any stages of chronic kidney disease, hypertensive kidney disease, glomerulonephritis, nephritic syndrome, hereditary nephropathy, end-stage renal disease, unspecified kidney failure, dialysis, and kidney transplant status.

### AI-enabled ECG classification performance

**Fig. 1:**
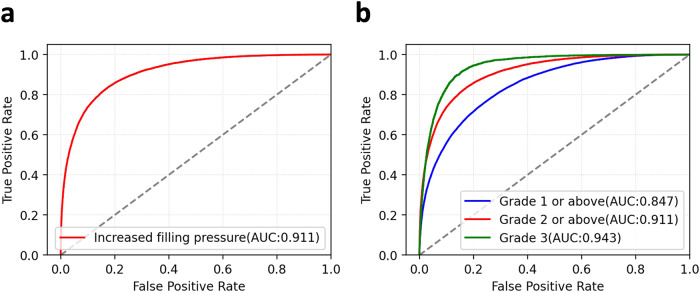
AI-enabled ECG ROC curves for diastolic function grade and filling pressure. **a** ROC plot for detecting increased filling pressure. **b** ROC plots for detecting diastolic function grades using an ordinal scale. ROC receiver operating characteristic, AUC area under the curve.

**Fig. 2:**
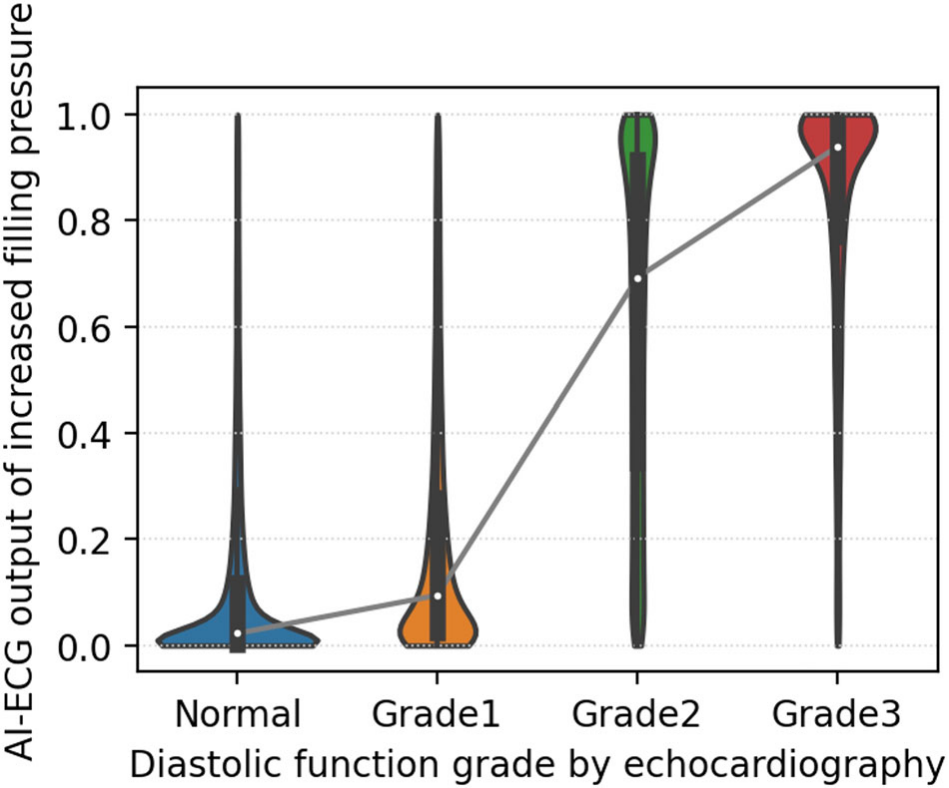
AI-enabled ECG output distribution for increased filling pressure by estimated diastolic function grade. The distribution was described as a box plot with a kernel density plot. Box plots show median and first and third quartiles with outliers as 1.5 times IQR.

In the test set, the AI-enabled ECG for predicting echocardiographically determined increased filling pressure had an area under the curve (AUC) of the receiver operating characteristic (ROC) curve of 0.911 (95% CI: 0.909–0.914) with a sensitivity of 83.2%, specificity of 82.9%, positive predictive value (PPV) of 58%, and negative predictive value (NPV) of 94.5% with the threshold of 0.26, and prevalence of 22.2% (Fig. 1a and Table 2). The AI-enabled ECG’s AUCs for grade ≥1, grade ≥2, and grade 3 were 0.847 (95% CI: 0.844–0.85), 0.911 (95% CI: 0.909–0.914), and 0.943 (95% CI: 0.938–0.948) at thresholds of 0.443, 0.264, 0.058, respectively (Fig. 1b and Table 2). The median output values for the increased filling pressure from the model were significantly higher in the diastolic function grades 2 and 3 by echocardiography compared to normal and grade 1 (Fig. 2). The model showed higher specificity in younger patients and among patients with more comorbidities there was a tendency towards decreased specificity (Supplemental Fig. 1 and Table 3). Echocardiographic diastolic parameters significantly differed between patients identified by the model to have increased and normal filling pressure patients in the testing (Supplemental Fig. 2). Those diastolic parameters were almost identical in patients with normal filling pressure determined by AI-ECG and echocardiography. Those values in patients with increased filling pressure by both AI-ECG and echocardiography were consistent with grade 2–3 diastolic dysfunction and significantly different from values in patients with normal filling pressure. In the indeterminate group, all echocardiographic diastolic parameters except e’ velocity are significantly different between normal and increased filling pressure determined by AI-ECG (Supplemental Fig. 3).

**Table 2:** Model performance for filling pressure and diastolic function from the AI-enabled ECG in test set with AUC, sensitivity, specificity, PPV, and NPV.

Class	Prevalence	AUC	Sensitivity (%)	Specificity (%)	PPV (%)	NPV (%)
Increased filling pressure	22.2% (21,883/98,763)	0.911	83.2	82.9	58.1	94.5
Grade 1 or above	42% (41,462/98,763)	0.847	76.6	75.3	69.2	81.6
Grade 2 or above	22.2% (21,883/98,763)	0.911	83.2	82.9	58.1	94.5
Grade 3	4.1% (4068/98,763)	0.943	89.8	86.0	21.6	99.5

Thresholds for grade 1 or above, grade 2 or above (increased filling pressure), and grade 3 are 0.443, 0.264, and 0.058, respectively, to evaluate the performance.

The AI-enabled ECG trained exclusively by ECG Lead I had AUCs of 0.804 (95% CI: 0.801–0.807), 0.875 (95% CI: 0.872–0.878), and 0.915 (95% CI: 0.909–0.921) for grade ≥1, grade ≥2, and grade 3, respectively. The AI-enabled ECG trained by ECG Lead I median beat had AUCs of 0.763 (95% CI: 0.76–0.766), 0.834 (95% CI: 0.83–0.837), and 0.877 (95% CI: 0.87–0.884), respectively.

The AUCs of AI-enabled ECG between before and after the median year of the echocardiography exam, i.e., 2014, were 0.856 and 0.839 for grade ≥1, 0.91 and 0.913 for grade ≥2, and 0.944 and 0.942 for grade 3, respectively (Supplemental Fig. 4).

### Survival analysis

**Fig. 3:**
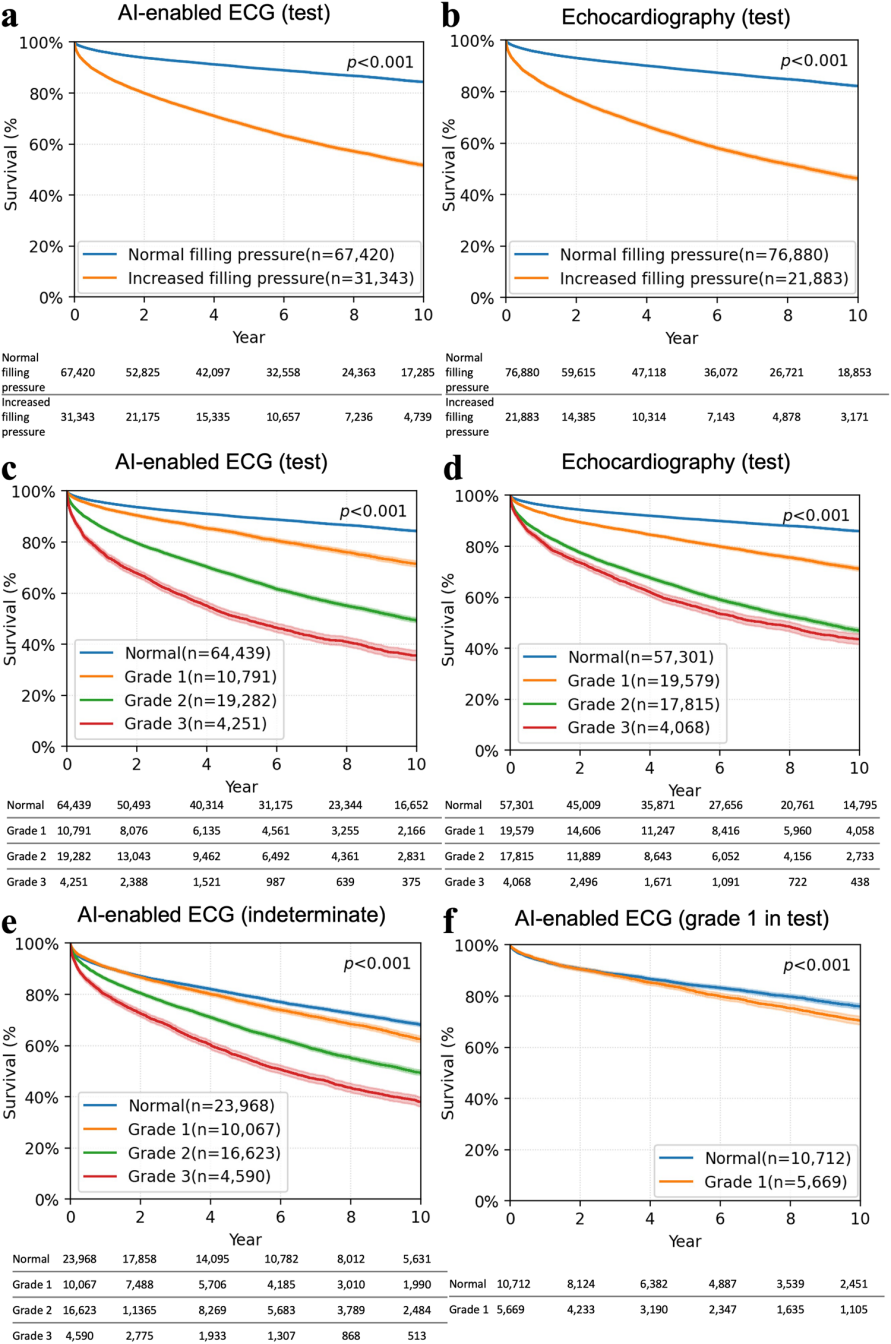
All-cause mortality using a Kaplan–Meier curve with 95% point-wise confidence intervals. **a** Kaplan–Meier curve for a test group of patients according to filling pressure predicted by the AI-enabled ECG. **b** Kaplan–Meier curve for a test group of patients according to filling pressure by echocardiography. **c** Kaplan–Meier curve for the test group according to diastolic function grades predicted by the AI-enabled ECG. **d** Kaplan–Meier curve according to diastolic function grades by echocardiography. **e** Kaplan–Meier curve for the indeterminate group according to diastolic function grades predicted by the deep learning model. **f** Kaplan–Meier curve for echocardiographic grade 1 in the testing group according to diastolic function grade normal and grade 1 predicted by the AI-enabled ECG. Number at risk tables are described below Kaplan–Meier curves. Log-rank test is used for the *p*-value. ECG electrocardiogram.

Death from any cause was observed in 20,223 (20.5%) of 98,763 patients in the test group and 18,224 (33.0%) of 55,248 in the indeterminate group over a median follow-up of 5.9 years (IQR 2.7, 10.2) and 5.7 years (IQR 2.6, 9.9), respectively. Mortality was significantly higher in patients with increased filling pressure compared to those with normal filling pressure predicted by the AI-enabled ECG after adjusting for age, sex, and comorbidities (hazard ratio (HR) 1.7, 95% CI 1.645–1.757; Fig. 3a). It was similar to the mortality predicted by echocardiographically determined filling pressure (HR 1.65, 95% CI: 1.597–1.705; Fig. 3b). All-cause mortality was also predicted by diastolic function grading determined by ECG with significantly higher mortality in patients with grade 2 and 3 diastolic dysfunction compared to those with normal or grade 1 diastolic dysfunction (HR 1.299, 95% CI 1.279–1.319, Fig. 3c). Diastolic function grading based directly upon echocardiographic parameters had a similar prognostic value (HR 1.298, 95% CI 1.277–1.32, Fig. 3d) even after adjusting for age, sex, and comorbidities. Since some patients had a discordance between AI-ECG and echocardiography determination of diastolic function, the study patients were separated into four groups in each category of diastolic dysfunction: true positive (TP; AI-ECG (+) and echocardiography (+), true negative (TN; AI-ECG (−) and echocardiography (−), false positive (FP; AI-ECG (+) and echocardiography (−), and false negative (FN; AI-ECG (−) and echocardiography (+)). While TP had the worst mortality and TN the best in all 3 diastolic dysfunction groups, FP and FN groups had a similar mortality in grade ≥1 or ≥2, but FP was found to have the same mortality as that of TP which was significantly worse than that of FN (HR 1.402, 95% CI 1.281–1.535) for grade 3 after adjusting for age, sex, and comorbidities (Supplemental Fig. 5).

The risk of death was also greater among patients in the indeterminate group with higher filling pressure predicted from the AI-enabled ECG (HR 1.34, 95% CI 1.298–1.383). Among patients with normal filling pressure by the AI-enabled ECG, grade 1 dysfunction had worse survival than normal grade in both the testing and the indeterminate groups (Fig. 3c, e). Among patients with grade 1 diastolic dysfunction by echocardiography, 54.7% were classified as normal by the AI-enabled ECG, and those who were labeled as normal had lower risk of death than patients who labeled as grade 1 dysfunction by the AI-enabled ECG (Fig. 3f). The AI-enabled ECG successfully discriminated risk of death among specific age groups (≤50 years, 50 < age < 70 years, age ≥ 70 years) even after adjusting for age, sex and comorbidities (Supplemental Fig. 6).

## Discussion

Here we report that a deep neural network of an AI-enabled ECG was developed to detect patients with increased filling pressure and to grade diastolic dysfunction. This study shows three main findings. First, our AI-enabled ECG model is able to identify patients with increased left ventricular filling pressure with high accuracy. Second, the prediction of the model for diastolic function and filling pressure is well aligned with echocardiographically determined diastolic function and estimated diastolic filling pressure. Additionally, ECG-based diastolic function grading and filling pressure assessment are associated with mortality.

Recently, several machine learning-based algorithms have been developed to predict echocardiographic diastolic dysfunction assessment^13–16^. Chao et al.^13^ used nine diastolic function variables to classify patients into normal, impaired relaxation and increased filling pressure groups. Pandey et al.^16^ developed a deep neural network algorithm to identify increased filling pressure based on clusters from an unsupervised learning network using echocardiographic values. Tromp et al.^17^ used a deep learning algorithm to automatically annotate 2D videos and Doppler velocities for diastolic function assessment. Gruca et al.^18^ used machine learning to train left atrial strain (LAS) curves to detect LV end-diastolic pressure ≥15 mmHg, yielding a LAS index. However, these studies relied upon echocardiographic and Doppler parameters to evaluate diastolic function. Kagiyama et al.^15^ suggested a machine learning model using ECG to estimate myocardial relaxation to detect diastolic dysfunction, but the model was not designed to predict diastolic filling pressure which is more closely associated with heart failure symptoms and prognosis. The present model was designed to predict filling pressure as well as diastolic function grade using only an ECG which is much more cost-effective and scalable to a wider range of population. It is noteworthy that echocardiographic parameters in patients with different diastolic function grades predicted by the AI-enabled ECG were comparable to those in the corresponding grades determined by echocardiography. Advanced diastolic dysfunction with increased filling pressure determined by echocardiography has been shown to predict increased mortality in various cardiovascular disorders including heart failure with preserved ejection fraction (HFpEF)^4,7,19^. Likewise, the AI-enabled ECG discriminated risk of death as well as the stratification based upon echocardiography. This is likely explained by characteristic ECG features shaped by delayed myocardial relaxation and increased filling pressure detected by the neural network. Tsai et al.^20^ have shown that AI-ECG is predictive of mortality in various cardiovascular diseases. Their deep learning model was based on ECG patterns associated with mortality and designed to identify high-risk patients due to any cardiovascular disease. Our model was predictive of mortality based on increased filling pressure. Since increased filling pressure is a key component of various cardiovascular diseases resulting in heart failure, our data suggest a promising role for AI-enabled ECG as a screening test to identify or exclude a cardiac cause in patients who present with dyspnea or other signs of heart failure. HFpEF is a common and growing public health problem that particularly affects older adults^2^. Clinical trials have recently demonstrated a significant benefit in heart failure with preserved ejection fraction patients from sodium–glucose Cotransporter-2 inhibitor^21,22^, but these treatments can only be administered when the diagnosis is established, and current evidence suggests that HFpEF remains under-recognized in the community^23,24^. Demonstration of increased left ventricular filling pressure is necessary to support the diagnosis of HFpEF, but in most cases requires either echocardiographic or even invasive evaluation^23,25^. The present AI-enabled ECG may be able to identify patients with a high likelihood of HFpEF in conjunction with AI-ECG determination of left ventricular ejection fraction. It may also be possible to identify relatively asymptomatic patients with early-stage HFpEF and they can be treated to avoid symptomatic heart failure. Prospective clinical trials will be required to prove such a potent, but hypothetical use of AI-ECG to detect patients with increased filling pressure. Another potential clinical application of AI-ECG for diastolic function with a high negative predictive value would be to exclude cardiac etiology in patients with unexplained dyspnea when the AI-enabled ECG indicates normal diastolic function or filling pressure. A preliminary retrospective data in two thousand patients with dyspnea evaluated in the emergency department at our medical center, AI-ECG determination of normal and increased filling pressure was shown to be significantly associated with non-cardiac and cardiac dyspnea, respectively (unpublished). In patients with known heart failure, this model may be able to monitor response to its management to optimize the filling pressure. Since the AI-enabled ECG trained by single-lead or median beat ECG showed a promising performance, it can be incorporated into the patient’s watch to adjust heart failure medications.

Echocardiography is the established diagnostic tool with well-documented reliability in the determination of diastolic function and filling pressure^6^ and was used as the reference technique in this study. Our data suggest that the AI-enabled ECG may also offer incremental value to echocardiographic assessment of diastolic function. The model can predict diastolic filling pressure when echocardiography is not able to assess diastolic function, which is a relatively frequent situation in clinical practice. Moreover, the risk of death was robustly predicted by our model in patients whose diastolic function assessment was indeterminate by echocardiography. Echocardiographic parameters reflecting diastolic function and filling pressure (the ratio between mitral inflow early diastolic velocity (*E*) and mitral annulus early diastolic velocity (*e*’) (*E*/*e*’), left atrial volume index, tricuspid regurgitation velocity, and the ratio between mitral inflow early and late velocity (*E*/*A*)) differed between normal and increased filling pressure predicted by ECG in the indeterminate group (Supplemental Fig. 3), similar to those in patients with clear echocardiographic determination of diastolic function. A main reason for indeterminate diastolic grading by echocardiography is a discrepancy among the four variables used for evaluating diastolic function. The recommended echocardiographic diastolic function parameters’ normal values are specific for diastolic dysfunction/increased filling pressures, but not sensitive^17,18^. The difference in echocardiographic diastolic parameters between the increased and normal filling pressure groups was smaller in the indeterminate than in the test group, which likely contributed to the discrepant echocardiographic evaluation.

It is noteworthy that 54.7% of the patients with grade 1 by echocardiography were identified as normal by our AI-enabled ECG. A main difference between normal and grade 1 dysfunction is delayed myocardial relaxation, but both have normal filling pressures. Grade 1 diastolic dysfunction by echocardiography includes a heterogeneous population. It is a common finding with normal senescence but is also observed in those with compensated heart failure. Interestingly, patients predicted as normal by our model were younger (66.1 ± 10.9 vs. 71.6 ± 9.1 years) and had fewer comorbidities compared to patients with grade 1 diastolic dysfunction by AI-ECG and echocardiography (Supplemental Table 4). Left atrial volume index, *e*’, *E*/*e*’, and *E*/*A* were significantly different (*p* < 0.001) between the two groups (Supplemental Fig. 7). Moreover, the group with grade 1 diastolic dysfunction by echocardiography and normal diastolic function by the present model had lower mortality than the group with grade 1 diastolic dysfunction by both echocardiography and the model. We have previously shown that one-third of the patients with grade 1 dysfunction at rest developed increased filling pressure with exercise^26,27^. It will be clinically helpful to investigate whether the filling pressure response to exercise is different between the two groups.

Our AI-ECG model has no exclusion criteria and the patients with reduced ejection fraction or cardiac diseases that are characterized by both diastolic dysfunction and abnormal ECG patterns such as hypertrophic cardiomyopathy, cardiac amyloidosis, and aortic stenosis were included. Interestingly, the majority of the patients with grade 3 diastolic dysfunction by AI-ECG had cardiac amyloidosis or reduced left ventricular ejection fraction followed by moderate to severe mitral regurgitation and hypertrophic cardiomyopathy (Supplemental Table 5). There were 9072 patients with low ejection fraction in the testing group and 3007 (33%) of them were predicted as having normal diastolic function or grade 1 by AI-ECG, but they had lower NPV compared to that of the patients with normal ejection fraction (Supplemental Fig. 8). Previously, we developed an AI-ECG for low EF^10^, defined as EF ≤ 35%, and the output of the AI-ECG EF showed AUC of 0.728 (95% CI: 0.724–0.733), sensitivity of 29.3%, and specificity of 95% for detecting increased filling pressure, indicating that AI-ECG models for low EF and diastolic filling pressure are based on different sets of ECG features. Supplemental Fig. 9 shows a case of a 30-year-old female who developed heart failure due to post-partum cardiomyopathy with LVEF of 30%. AI-ECG at that time showed reduced LVEF and increased filling pressure. She became asymptomatic with heart failure treatment, but LVEF remained reduced. AI-ECG at follow-up showed reduced LVEF and normal LV filling pressure.

There are several limitations to our study. First, this study used echocardiographic determination, non-invasive measure of diastolic function and filling pressure as their reference. While invasively measured filling pressure is the gold standard, echocardiographic measurement is a clinically accepted tool for estimating left ventricular filling pressure and has been adopted in the guideline and scoring algorithms for detecting heart failure with preserved ejection fraction^25,28,29^. In addition, only four echocardiographic parameters were used to determine diastolic function in this study. If more parameters were used such as pulmonary vein Doppler velocity, isovolumic relaxation time, and deceleration time of mitral inflow velocity, the diastolic function could have been determined in some patients with indeterminate diastolic function. However, most of the clinical practice relies on those four parameters. Second, ECG and echocardiography were obtained non-simultaneously, but the mean interval between the two tests was 0.5 ± 1.2 days. This limitation may explain some discrepancies between the AI-enabled ECG and echocardiography of filling pressure. However, the progression of diastolic function status takes a long time, and it is very unlikely to have a major change in filling pressure in 2 weeks. Third, our AI-ECG model was created from retrospective data. While there are numerous potential clinical applications of this model, future prospective studies are needed to establish the precise clinical diagnostic and prognostic role of our model in various cardiovascular diseases. Fourth, the data were only collected at a major tertiary medical center in the United States, so external validations in more heterogeneous populations with greater diversity are needed. Fifth, our current model has limited interpretability to elucidate the prediction of diastolic function grade besides heatmap-based approaches. To enhance interpretability, explainable AI models trained leveraging ECG features for diastolic function become imperative.

In conclusion, the present study shows that the application of AI-enabled ECG allows for grading of diastolic dysfunction and estimation of filling pressure, with a robust prognostic value similar to that of comprehensive echocardiography. These data suggest that AI-enabled ECG may be useful to enhance diagnostic evaluation of disorders associated with diastolic dysfunction and increased filling pressure in the community, including heart failure with preserved ejection fraction.

## Methods

### Data and study population

We identified all adults (age ≥18 years) who had at least one ECG and transthoracic echocardiogram with echocardiographic assessment of diastolic function performed within 14 days of ECG between September 2001 and April 2023 from the Mayo Clinic Unified Data Platform. No exclusion criteria were applied. All ECGs were measured with 250 or 500 Hz sampling rate using a GE-Marquette machine for a standard 10-s 12-lead ECG and were stored in the GE-MUSE system (Marquette, WI, USA). ECGs with an original sampling rate of 250 Hz were up-sampled to 500 Hz prior to analysis. The final cohort (*n* = 219,462) was divided into training (*n* = 98,736, 45%), validation (*n* = 21,963, 10%), and testing (*n* = 98,763, 45%) sets. We further tested our final model in 55,248 patients with indeterminate diastolic function by echocardiography (for additional details on the cohort, see Supplemental Fig. 10). The Mayo Clinic Internal Review Board (Jeche, Resa Jo) approved this study with a waiver of the requirement to obtain informed consent in accordance with 45 CFR 46.104d and a waiver of Health Insurance Portability and Accountability Act (HIPAA) authorization in accordance with applicable HIPAA regulations.

### Filling pressure and diastolic function grading assessment

As recommended by the 2016 ASE/EACVI diastolic function guidelines^8^, four parameters were used to evaluate diastolic function: *e*’, *E*/*e*’, tricuspid regurgitation velocity, and left atrial volume index, with a minor modification^30^ (Supplemental Fig. 11). When 3 or all of the above four parameters were abnormal, filling pressure was determined to be elevated. This group was subsequently separated into grade 2 or 3 based on *E*/*A* of 2.0. When 3 or all parameters were normal, filling pressure was determined to be normal. These patients were further separated into normal diastolic function or grade 1 according to the *E*/*A* ratio separating the value of 0.8. When the four parameters were split into 2 normal and 2 abnormal, diastolic function was assessed as indeterminate. Diastolic function and filling pressure were labeled based on the above algorithms for all subjects.

### Overview of AI model

The primary goal of developing the AI-enabled ECG was to predict left ventricular filling pressure and diastolic function grade using a 12-lead ECG. As model architecture, we implemented convolutional neural networks of the ResNet-18^31^. Each ECG has 12 × 5000 matrix that consists of 12-lead ECG by 10-s sampled at 500 Hz. For input of the network, we split the ECG by 2 s and average the output values from 5 splits. The network was trained with a learning rate of 0.001 and Adam optimizer for 20 epochs. The validation performance was converged before the 20th epoch. The final model was chosen according to the AUC value from the validation set for increased filling pressure. The model was trained as a multi-class model with four outputs representing the four grades of diastolic function and the sum of four outputs was 1. Normal and grade 1 were considered normal filling pressure, and grades 2 and 3 were considered increased filling pressure. While the model outputs four values, the sum of the outputs of normal and grade 1 represents the output of normal filling pressure, and the sum of grades 2 and 3 outputs represents the output of increased filling pressure. Likewise, the sum of the outputs of normal and increased filling pressures was 1. Using the sum of each two classes, we converted the multi-class model to a binary model and we applied the Youden index^32^ to the final output value. Likewise, we created an aggregated output and a label for grade 1 or above, grade 2 or above, and grade 3, respectively, to evaluate the performance with the ordinal scale of diastolic function grade. Additionally, we trained deep neural networks with the same architecture for single-lead ECG and single-lead median beat. Since Lead I is most widely measured for wearable devices, Lead I is used for both models. Single-lead median beat is the representative beat for 10-s and the duration is 1.2 s. We reported the hold-out test set result from the selected model.

### Statistical analysis

The model’s ability was assessed by calculating the AUC of the ROC curve, sensitivity, specificity, PPV, NPV, and accuracy. Two-sided 95% confidence intervals were calculated. We also assessed whether the model discriminates the risk of all-cause mortality using the Kaplan–Meier estimate and compared it with the log-rank test. Multivariable Cox proportional-hazards models were developed. Age, sex, and comorbidities (diabetes, hypertension, obesity, myocardial infarction, congestive heart failure, cerebrovascular disease, chronic pulmonary disease, and renal disease) were used for the adjustment of the hazard ratio. For continuous variables, groups were compared using Student’s *t*-test. For categorical variables, chi-squared tests were used. A two-tailed *P*-value < 0.001 was considered significant, however, its interpretation was carefully and comprehensively made because of the large sample size.

### Supplementary information

Supplemental video

Supplemental material

## Data Availability

All requests for raw and analyzed data and related materials, excluding programming code, will be reviewed by the Mayo Clinic legal department and Mayo Clinic Ventures to verify whether the request is subject to any intellectual property or confidentiality obligations. Requests for patient-related data not included in the paper will not be considered. Any data and materials that can be shared will be released via a Material Transfer Agreement.

## Code availability

Any custom code or software used to implement the deep learning model detailed in this paper will be made available upon request.
