# Supplementary material for: Artificial intelligence-enabled ECG for left ventricular diastolic function and filling pressure
Source: NPJ Digit Med. 2024 Jan 6;7:4. doi: 10.1038/s41746-023-00993-7 (PMC10770308; doi:10.1038/s41746-023-00993-7)
Supplement: Supplementary file 2 — Supplemental material [file 41746_2023_993_MOESM2_ESM.pdf]

Supplemental Material

Table of contents

1. Figure

1. AUC, sensitivity, specificity, PPV, NPV, and odd ratio (OR) with 95% confidence intervals across age, gender, and comorbidity subsets. a. grade 1 or above. b. grade 2 or above (increased filling pressure) c. grade 3.

2. The distribution of left atrial volume index, e', E/e', tricuspid regurgitation velocity, and E/A according to filling pressure by our AI-ECG and echocardiography in test set.

3. The distribution of left atrial volume index, e', E/e', tricuspid regurgitation velocity, and E/A according to filling pressure by our AI-ECG in indeterminate set.

4. The ROC curves of AI-ECG between before and after the median year of the echocardiography exam in the test set.

5. All-cause mortality using a Kaplan-Meier curve with 95% confidence intervals and p-value from a log-rank test by true positive (TP), false positive (FP), false negative (FN), and true negative (TN) for grade 1 or above, grade 2 or above, and grade 3.

6. All-cause mortality using a Kaplan-Meier curve with 95% confidence intervals and p-value from a log-rank test by age. a. Kaplan-Meier curve for test group of patients (age≤ 50) according to the filling pressure by our AI-ECG (HR 1.413, 95% CI 1.329-1.503). b. Kaplan-Meier curve for test group of patients (50< age <70) according to the filling pressure by our AI-ECG (HR 1.35, 95% CI 1.313-1.388). c. Kaplan-Meier curve for test group of patients (age≥70) according to the filling pressure by our AI-ECG (HR 1.235, 95% CI 1.211-1.26).

7. The distribution of left atrial volume index, e', E/e', tricuspid regurgitation velocity, and E/A according to diastolic filling pressure by our AI-ECG in grade 1 by echocardiography.

8. The ROC curves and performance of AI-ECG in normal ejection fraction and low ejection fraction, defined as left ventricular ejection fraction <50% in the test set.

9. An illustrative case of a heart failure patient.

10. Flow chart of dataset construction and patient distribution. a. A flow chart demonstrating patient selection. b. A pie chart for dataset split.

11. Algorithm for assessment of diastolic filling pressure and function.

2. Table

1. Patient characteristics of training, validation, and testing group.

2. Patient characteristics of four diastolic grade groups determined by AI-ECG in test set.

3. Model performance for filling pressure and diastolic function grade from AI-ECG in test set by hypertension, obesity, and diabetes.

4. Clinical characteristics of normal or grade 1 group by AI-ECG in grade 1 patients by echocardiography.

5. The number of patients for hypertrophic cardiomyopathy (HCM), cardiac amyloidosis, moderate to severe mitral valve regurgitation (MR) and aortic stenosis (AS), and reduced left ventricular ejection fraction (LVEF) according to diastolic function grade by AI-ECG.

1. **Figure**

Figure 1. AUC, sensitivity, specificity, PPV, NPV, and odd ratio (OR) with 95% confidence intervals across age, gender, and comorbidity subsets. a. grade 1 or above. b. grade 2 or above (increased filling pressure) c. grade 3.

a.

Group	Positive / Total (Prevalence)	AUC (95% CI)	Sensitivity (95% CI)	Specificity (95% CI)	PPV (95% CI)	NPV (95% CI)	OR (95% CI)
Female Age<30	76/3012 (2.5%)	0.889 (0.842-0.936)	56.6% (45.4%-67.1%)	97.1% (96.4%-97.7%)	33.6% (26.0%-42.1%)	98.9% (98.4%-99.2%)	43.71 (26.45-72.22)
Male Age<30	86/2441 (3.5%)	0.906 (0.863-0.948)	61.6% (51.1%-71.2%)	97.1% (96.4%-97.7%)	43.8% (35.3%-52.7%)	98.6% (98.0%-99.0%)	54.02 (32.86-88.80)
Female Ages 30-39	205/3639 (5.6%)	0.867 (0.840-0.895)	57.1% (50.2%-63.7%)	95.3% (94.5%-95.9%)	41.8% (36.2%-47.6%)	97.4% (96.8%-97.9%)	26.68 (19.41-36.68)
Male Ages 30-39	245/2663 (9.2%)	0.899 (0.876-0.921)	66.5% (60.4%-72.1%)	94.4% (93.4%-95.3%)	54.7% (49.0%-60.3%)	96.5% (95.7%-97.2%)	33.62 (24.48-46.16)
Female Ages 40-49	675/5682 (11.9%)	0.824 (0.806-0.842)	53.6% (49.9%-57.4%)	90.7% (89.8%-91.4%)	43.7% (40.3%-47.1%)	93.6% (92.8%-94.2%)	11.24 (9.40-13.44)
Male Ages 40-49	702/5078 (13.8%)	0.816 (0.798-0.834)	57.3% (53.6%-60.9%)	88.6% (87.6%-89.5%)	44.7% (41.4%-47.9%)	92.8% (92.0%-93.6%)	10.43 (8.75-12.45)
Female Ages 50-59	2366/9104 (26.0%)	0.763 (0.751-0.774)	54.1% (52.0%-56.1%)	81.8% (80.8%-82.7%)	51.0% (49.0%-53.0%)	83.5% (82.6%-84.4%)	5.27 (4.76-5.84)
Male Ages 50-59	2378/9235 (25.7%)	0.779 (0.768-0.790)	59.8% (57.9%-61.8%)	79.9% (79.0%-80.9%)	50.8% (49.0%-52.7%)	85.2% (84.3%-86.0%)	5.94 (5.36-6.57)
Female Ages 60-69	5175/11529 (44.9%)	0.742 (0.733-0.751)	68.0% (66.7%-69.2%)	66.1% (64.9%-67.2%)	62.0% (60.7%-63.3%)	71.7% (70.5%-72.8%)	4.13 (3.82-4.47)
Male Ages 60-69	5295/12496 (42.4%)	0.745 (0.737-0.754)	68.7% (67.4%-69.9%)	65.9% (64.8%-67.0%)	59.7% (58.4%-60.9%)	74.1% (73.0%-75.1%)	4.23 (3.92-4.56)
Female Ages 70-79	6736/10180 (66.2%)	0.724 (0.714-0.734)	81.3% (80.3%-82.2%)	45.2% (43.5%-46.8%)	74.3% (73.3%-75.3%)	55.2% (53.3%-57.0%)	3.57 (3.26-3.91)
Male Ages 70-79	6873/10920 (62.9%)	0.733 (0.724-0.743)	80.5% (79.5%-81.4%)	47.7% (46.2%-49.3%)	72.3% (71.3%-73.3%)	59.0% (57.3%-60.7%)	3.77 (3.46-4.11)
Female Age>=80	5987/6947 (86.2%)	0.751 (0.736-0.767)	92.5% (91.8%-93.1%)	28.2% (25.5%-31.2%)	88.9% (88.1%-89.7%)	37.5% (34.0%-41.1%)	4.82 (4.06-5.71)
Male Age>=80	4663/5836 (79.9%)	0.731 (0.716-0.746)	90.6% (89.7%-91.4%)	29.2% (26.6%-31.8%)	83.6% (82.5%-84.6%)	43.8% (40.4%-47.4%)	3.97 (3.38-4.66)
Negative for Cerebrovascular Disease	36023/89336 (40.3%)	0.847 (0.845-0.850)	75.6% (75.2%-76.1%)	76.3% (75.9%-76.6%)	68.3% (67.8%-68.7%)	82.2% (81.9%-82.6%)	9.97 (9.67-10.29)
Positive for Cerebrovascular Disease	5342/9177 (58.2%)	0.820 (0.811-0.828)	83.3% (82.3%-84.3%)	61.9% (60.4%-63.4%)	75.3% (74.2%-76.4%)	72.7% (71.1%-74.2%)	8.11 (7.36-8.93)
Negative for Chronic Pulmonary Disease	32847/83551 (39.3%)	0.846 (0.844-0.849)	75.4% (74.9%-75.9%)	76.3% (75.9%-76.7%)	67.3% (66.9%-67.8%)	82.7% (82.4%-83.1%)	9.88 (9.57-10.21)
Positive for Chronic Pulmonary Disease	8518/14962 (56.9%)	0.834 (0.828-0.841)	81.3% (80.4%-82.1%)	67.3% (66.2%-68.5%)	76.7% (75.8%-77.6%)	73.1% (72.0%-74.3%)	8.96 (8.31-9.66)
Negative for Congestive Heart Failure	29357/81731 (35.9%)	0.827 (0.824-0.830)	71.0% (70.5%-71.5%)	77.1% (76.7%-77.5%)	63.5% (62.9%-64.0%)	82.6% (82.2%-82.9%)	8.24 (7.97-8.51)
Positive for Congestive Heart Failure	12008/16782 (71.6%)	0.849 (0.842-0.855)	90.4% (89.9%-90.9%)	55.7% (54.2%-57.1%)	83.7% (83.0%-84.3%)	69.7% (68.3%-71.2%)	11.82 (10.87-12.84)
Negative for Diabetes	31814/83337 (38.2%)	0.846 (0.843-0.849)	74.3% (73.9%-74.8%)	77.3% (76.9%-77.6%)	66.9% (66.4%-67.4%)	83.0% (82.6%-83.3%)	9.85 (9.54-10.18)
Positive for Diabetes	9648/15426 (62.5%)	0.809 (0.802-0.816)	84.1% (83.3%-84.8%)	57.6% (56.3%-58.9%)	76.8% (76.0%-77.6%)	68.4% (67.1%-69.7%)	7.16 (6.64-7.72)
Negative for Hypertension, combined	16218/53693 (30.2%)	0.852 (0.849-0.856)	69.7% (69.0%-70.4%)	82.1% (81.7%-82.5%)	62.8% (62.1%-63.5%)	86.2% (85.9%-86.6%)	10.55 (10.11-11.01)
Positive for Hypertension, combined	25147/44820 (56.1%)	0.809 (0.805-0.813)	81.1% (80.6%-81.6%)	62.3% (61.6%-63.0%)	73.3% (72.8%-73.9%)	72.1% (71.4%-72.7%)	7.10 (6.80-7.41)
Negative for Myocardial Infarction	36179/90058 (40.2%)	0.846 (0.843-0.848)	75.2% (74.8%-75.7%)	76.4% (76.0%-76.7%)	68.1% (67.7%-68.6%)	82.1% (81.8%-82.4%)	9.82 (9.52-10.13)
Positive for Myocardial Infarction	5186/8455 (61.3%)	0.828 (0.819-0.837)	86.4% (85.5%-87.3%)	57.4% (55.7%-59.1%)	76.3% (75.2%-77.4%)	72.7% (71.0%-74.4%)	8.58 (7.73-9.54)
Negative for Obesity	27021/66174 (40.8%)	0.860 (0.857-0.863)	76.2% (75.7%-76.8%)	78.0% (77.5%-78.4%)	70.5% (70.0%-71.0%)	82.6% (82.2%-83.0%)	11.36 (10.95-11.78)
Positive for Obesity	14438/32579 (44.3%)	0.819 (0.815-0.824)	77.3% (76.6%-78.0%)	69.5% (68.8%-70.2%)	66.9% (66.1%-67.6%)	79.4% (78.7%-80.0%)	7.76 (7.38-8.16)
Negative for Renal Disease	32038/84400 (38.0%)	0.840 (0.837-0.842)	73.5% (73.0%-74.0%)	76.8% (76.5%-77.2%)	66.0% (65.5%-66.5%)	82.6% (82.3%-82.9%)	9.21 (8.92-9.51)
Positive for Renal Disease	9327/14113 (66.1%)	0.835 (0.828-0.842)	87.2% (86.5%-87.9%)	58.7% (57.2%-60.0%)	80.4% (79.7%-81.2%)	70.2% (68.8%-71.6%)	9.69 (8.91-10.54)
Overall	41462/98763 (42.0%)	0.847 (0.845-0.849)	76.6% (76.2%-77.0%)	75.3% (74.9%-75.6%)	69.2% (68.7%-69.6%)	81.6% (81.3%-82.0%)	9.98 (9.68-10.28)

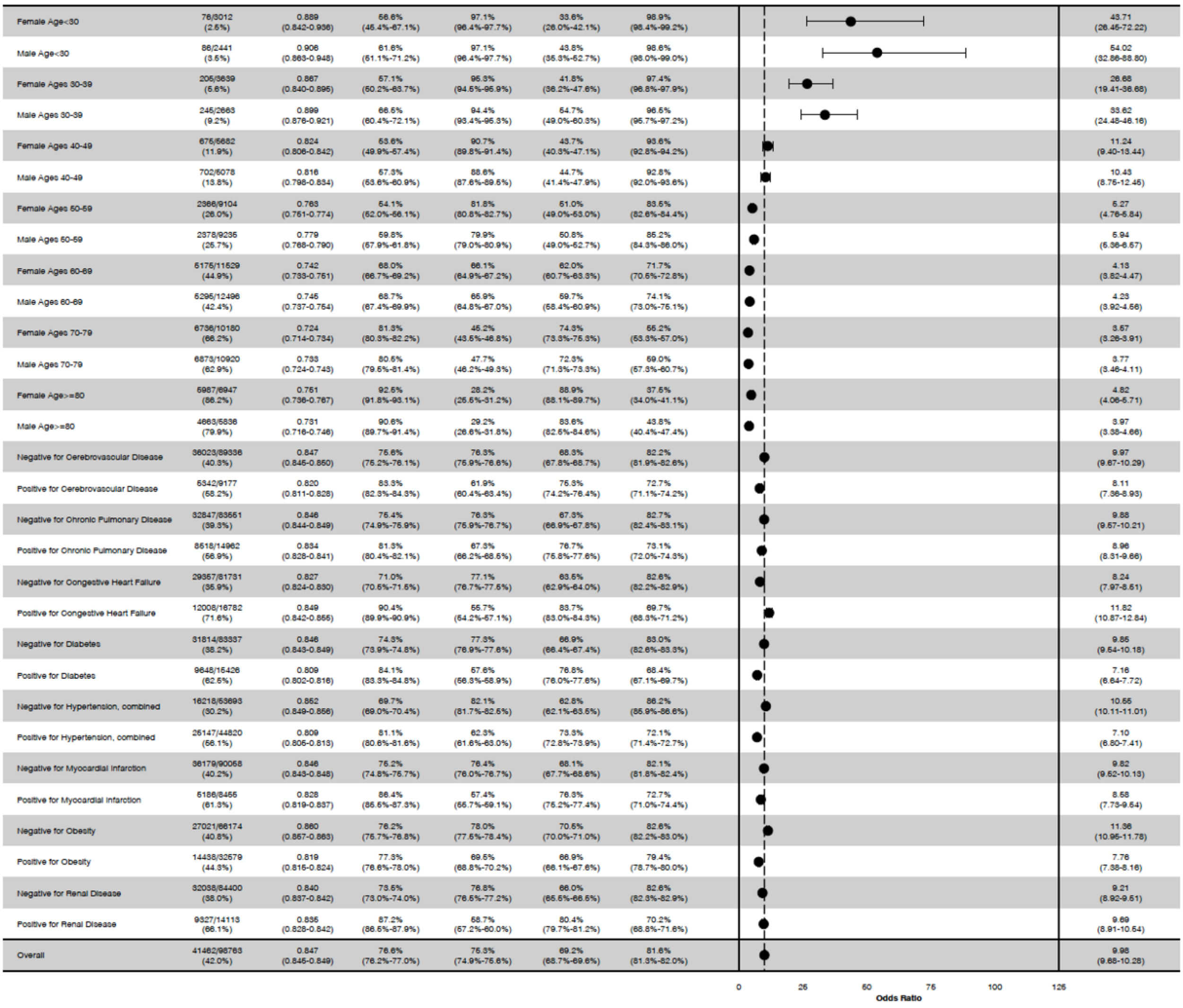

b.

Group	Positive / Total (Prevalence)	AUC (95% CI)	Sensitivity (95% CI)	Specificity (95% CI)	PPV (95% CI)	NPV (95% CI)	OR (95% CI)
Female Age<30	54/3014 (1.8%)	0.956 (0.921-0.991)	74.1% (61.1%-83.9%)	97.5% (96.8%-98.0%)	34.8% (26.7%-43.9%)	99.5% (99.2%-99.7%)	109.90 (57.35-210.61)
Male Age<30	67/2439 (2.7%)	0.968 (0.948-0.988)	79.1% (67.9%-87.1%)	96.9% (96.1%-97.5%)	41.7% (33.5%-50.4%)	99.4% (99.0%-99.6%)	117.56 (62.44-221.36)
Female Ages 30-39	125/3634 (3.4%)	0.965 (0.950-0.979)	81.6% (73.9%-87.4%)	96.1% (95.4%-96.7%)	42.7% (36.6%-49.0%)	99.3% (99.0%-99.5%)	109.15 (67.30-177.04)
Male Ages 30-39	166/2668 (6.2%)	0.971 (0.962-0.981)	84.3% (78.0%-89.1%)	94.7% (93.8%-95.5%)	51.5% (45.6%-57.3%)	98.9% (98.4%-99.3%)	96.68 (61.41-152.20)
Female Ages 40-49	348/5677 (6.1%)	0.932 (0.917-0.948)	77.0% (72.3%-81.1%)	94.3% (93.6%-94.9%)	46.9% (42.8%-50.9%)	98.4% (98.1%-98.7%)	55.37 (42.05-72.92)
Male Ages 40-49	382/5083 (7.5%)	0.949 (0.938-0.960)	83.2% (79.2%-86.7%)	92.2% (91.4%-92.9%)	46.4% (42.7%-50.2%)	98.5% (98.1%-98.9%)	58.68 (43.95-78.33)
Female Ages 50-59	955/9091 (10.5%)	0.902 (0.891-0.914)	73.3% (70.4%-76.0%)	90.7% (90.1%-91.3%)	48.1% (45.5%-50.6%)	96.7% (96.2%-97.0%)	26.80 (22.80-31.50)
Male Ages 50-59	1076/9248 (11.6%)	0.926 (0.917-0.935)	82.1% (79.7%-84.2%)	88.5% (87.8%-89.2%)	48.4% (46.1%-50.7%)	97.4% (97.0%-97.7%)	35.20 (29.70-41.72)
Female Ages 60-69	2085/11528 (18.1%)	0.873 (0.864-0.882)	75.8% (73.9%-77.6%)	82.4% (81.6%-83.2%)	48.8% (47.0%-50.5%)	93.9% (93.4%-94.4%)	14.70 (13.12-16.46)
Male Ages 60-69	2245/12497 (18.0%)	0.898 (0.891-0.906)	81.4% (79.8%-83.0%)	82.7% (81.9%-83.4%)	50.7% (49.1%-52.4%)	95.3% (94.9%-95.7%)	20.94 (18.60-23.56)
Female Ages 70-79	3548/10175 (34.9%)	0.831 (0.823-0.839)	80.9% (79.6%-82.2%)	68.2% (67.1%-69.3%)	57.7% (56.3%-59.0%)	87.0% (86.0%-87.9%)	9.10 (8.24-10.04)
Male Ages 70-79	3426/10925 (31.4%)	0.861 (0.853-0.869)	83.7% (82.5%-84.9%)	71.0% (69.9%-72.0%)	56.9% (55.5%-58.2%)	90.5% (89.8%-91.2%)	12.59 (11.35-13.97)
Female Age>=80	4413/6940 (63.6%)	0.799 (0.788-0.809)	88.8% (87.8%-89.7%)	47.6% (45.6%-49.5%)	74.7% (73.5%-75.9%)	70.9% (68.7%-73.0%)	7.20 (6.37-8.13)
Male Age>=80	2993/5843 (51.2%)	0.834 (0.824-0.844)	87.8% (86.6%-89.0%)	54.0% (52.1%-55.8%)	66.7% (65.2%-68.2%)	80.9% (79.0%-82.6%)	8.47 (7.42-9.66)
Negative for Cerebrovascular Disease	18828/89336 (21.1%)	0.914 (0.911-0.916)	82.8% (82.3%-83.3%)	83.8% (83.5%-84.1%)	57.7% (57.1%-58.3%)	94.8% (94.6%-95.0%)	24.89 (23.84-25.98)
Positive for Cerebrovascular Disease	3002/9177 (32.7%)	0.882 (0.875-0.890)	85.6% (84.3%-86.8%)	72.8% (71.7%-73.9%)	60.5% (59.0%-62.0%)	91.2% (90.4%-92.0%)	15.96 (14.20-17.92)
Negative for Chronic Pulmonary Disease	16409/83551 (19.6%)	0.913 (0.911-0.915)	82.7% (82.1%-83.3%)	83.7% (83.4%-84.0%)	55.3% (54.7%-55.9%)	95.2% (95.0%-95.4%)	24.50 (23.41-25.63)
Positive for Chronic Pulmonary Disease	5421/14962 (36.2%)	0.894 (0.889-0.900)	84.7% (83.7%-85.6%)	77.6% (76.7%-78.4%)	68.2% (67.1%-69.3%)	89.9% (89.2%-90.5%)	19.06 (17.46-20.82)
Negative for Congestive Heart Failure	12420/81731 (15.2%)	0.892 (0.889-0.895)	76.0% (75.2%-76.7%)	85.1% (84.8%-85.3%)	47.7% (47.0%-48.4%)	95.2% (95.0%-95.4%)	18.02 (17.21-18.88)
Positive for Congestive Heart Failure	9410/16782 (56.1%)	0.890 (0.885-0.895)	92.7% (92.1%-93.2%)	62.6% (61.5%-63.7%)	76.0% (75.2%-76.8%)	87.0% (86.1%-87.9%)	21.26 (19.41-23.28)
Negative for Diabetes	15591/83337 (18.7%)	0.912 (0.910-0.914)	81.5% (80.9%-82.1%)	84.6% (84.3%-84.9%)	54.9% (54.3%-55.5%)	95.2% (95.0%-95.4%)	24.23 (23.15-25.36)
Positive for Diabetes	6292/15426 (40.8%)	0.882 (0.876-0.887)	87.2% (86.4%-88.0%)	70.5% (69.5%-71.4%)	67.0% (66.0%-68.0%)	88.9% (88.2%-89.6%)	16.30 (14.95-17.77)
Negative for Hypertension, combined	7462/53693 (13.9%)	0.926 (0.922-0.929)	80.0% (79.1%-80.9%)	88.4% (88.1%-88.6%)	52.6% (51.7%-53.5%)	96.5% (96.3%-96.6%)	30.38 (28.51-32.37)
Positive for Hypertension, combined	14368/44820 (32.1%)	0.883 (0.879-0.886)	84.8% (84.2%-85.4%)	74.7% (74.2%-75.1%)	61.2% (60.5%-61.9%)	91.2% (90.9%-91.6%)	16.47 (15.63-17.35)
Negative for Myocardial Infarction	18379/90058 (20.4%)	0.911 (0.909-0.913)	82.1% (81.6%-82.7%)	83.8% (83.6%-84.1%)	56.6% (56.0%-57.2%)	94.8% (94.6%-95.0%)	23.83 (22.83-24.87)
Positive for Myocardial Infarction	3451/8455 (40.8%)	0.889 (0.882-0.896)	88.8% (87.7%-89.9%)	69.6% (68.4%-70.9%)	66.9% (65.5%-68.2%)	90.1% (89.1%-91.0%)	18.27 (16.17-20.64)
Negative for Obesity	14074/66174 (21.3%)	0.919 (0.916-0.921)	83.8% (83.2%-84.4%)	83.8% (83.5%-84.2%)	58.4% (57.7%-59.0%)	95.0% (94.8%-95.2%)	26.90 (25.57-28.30)
Positive for Obesity	7808/32579 (24.0%)	0.896 (0.892-0.900)	82.0% (81.1%-82.8%)	80.9% (80.4%-81.4%)	57.6% (56.6%-58.5%)	93.4% (93.1%-93.8%)	19.32 (18.09-20.64)
Negative for Renal Disease	15136/84400 (17.9%)	0.907 (0.905-0.910)	80.3% (79.7%-81.0%)	84.5% (84.2%-84.8%)	53.1% (52.5%-53.8%)	95.2% (95.0%-95.3%)	22.29 (21.31-23.32)
Positive for Renal Disease	6694/14113 (47.4%)	0.886 (0.881-0.892)	89.6% (88.9%-90.3%)	67.9% (66.9%-69.0%)	71.6% (70.6%-72.6%)	87.9% (87.0%-88.7%)	18.33 (16.71-20.10)
Overall	21883/98763 (22.2%)	0.911 (0.909-0.913)	83.2% (82.7%-83.7%)	82.9% (82.6%-83.2%)	58.1% (57.5%-58.6%)	94.5% (94.4%-94.7%)	23.97 (23.03-24.96)

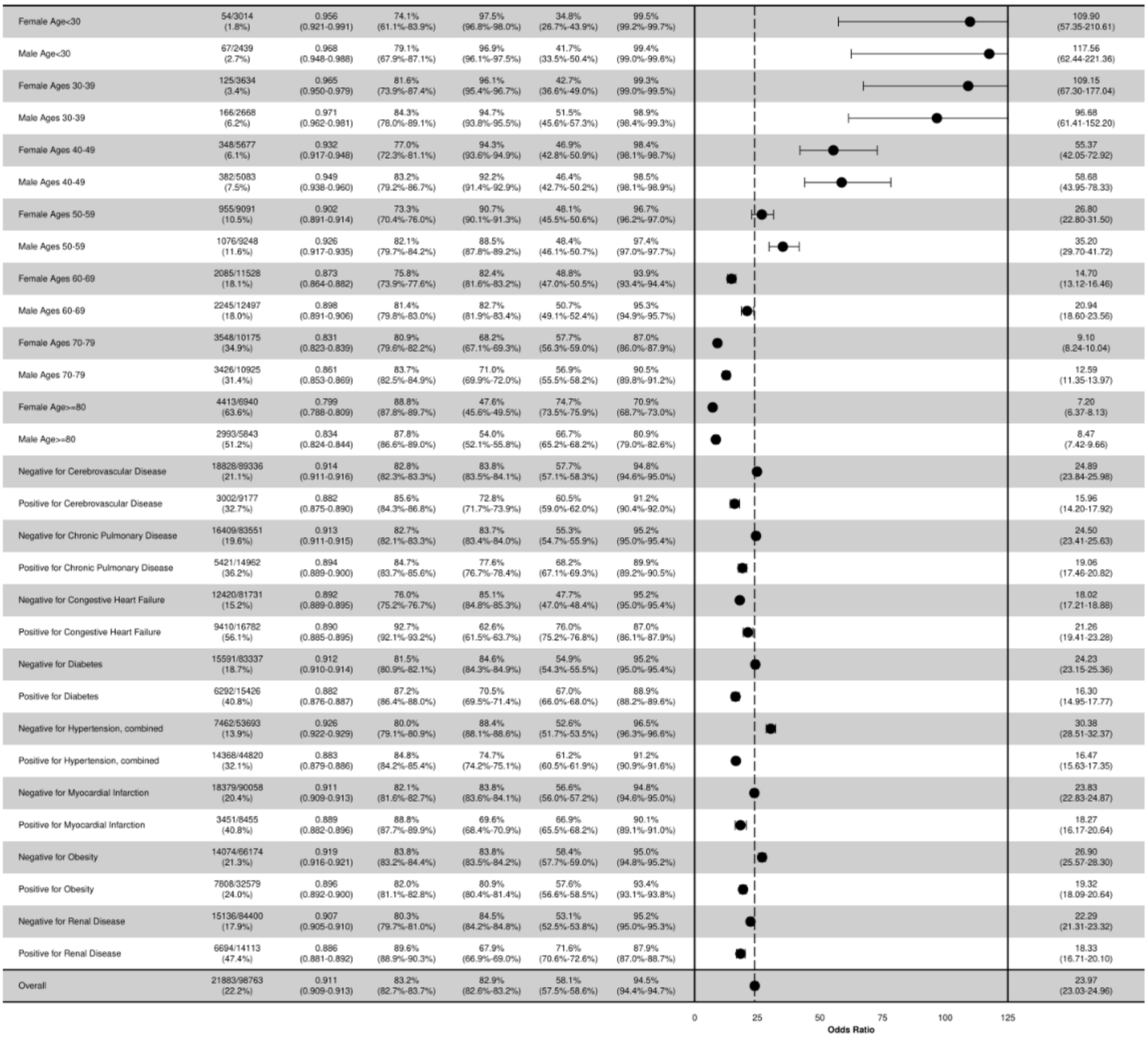

c.

Group	Positive / Total (Prevalence)	AUC (95% CI)	Sensitivity (95% CI)	Specificity (95% CI)	PPV (95% CI)	NPV (95% CI)	OR (95% CI)
Female Age<30	23/3012 (0.8%)	0.965 (0.928-1.000)	82.6% (62.9%-93.0%)	97.8% (97.2%-98.3%)	22.6% (15.0%-32.6%)	99.9% (99.6%-99.9%)	213.68 (70.71-645.72)
Male Age<30	34/2441 (1.4%)	0.959 (0.923-0.994)	82.4% (66.5%-91.7%)	97.1% (96.3%-97.7%)	28.6% (20.6%-38.2%)	99.7% (99.4%-99.9%)	155.80 (62.51-388.31)
Female Ages 30-39	44/3639 (1.2%)	0.984 (0.976-0.991)	88.6% (76.0%-95.0%)	96.3% (95.6%-96.8%)	22.5% (17.0%-29.3%)	99.9% (99.7%-99.9%)	201.46 (78.16-519.30)
Male Ages 30-39	70/2663 (2.6%)	0.970 (0.956-0.983)	88.6% (79.0%-94.1%)	94.7% (93.7%-95.5%)	31.0% (25.0%-37.7%)	99.7% (99.4%-99.8%)	137.87 (64.74-293.64)
Female Ages 40-49	90/5682 (1.6%)	0.960 (0.940-0.979)	84.4% (75.6%-90.5%)	95.5% (94.9%-96.0%)	23.2% (18.9%-28.0%)	99.7% (99.6%-99.8%)	115.03 (64.16-206.25)
Male Ages 40-49	149/5078 (2.9%)	0.977 (0.968-0.986)	94.6% (89.8%-97.3%)	93.6% (92.8%-94.2%)	30.8% (26.7%-35.2%)	99.8% (99.7%-99.9%)	256.42 (124.64-527.54)
Female Ages 50-59	156/9104 (1.7%)	0.961 (0.947-0.976)	89.1% (83.2%-93.1%)	92.9% (92.3%-93.4%)	17.9% (15.4%-20.8%)	99.8% (99.7%-99.9%)	106.86 (64.17-177.96)
Male Ages 50-59	373/9235 (4.0%)	0.957 (0.948-0.965)	89.0% (85.4%-91.8%)	90.7% (90.1%-91.3%)	28.7% (26.2%-31.4%)	99.5% (99.3%-99.6%)	78.88 (56.58-109.98)
Female Ages 60-69	279/11529 (2.4%)	0.940 (0.929-0.952)	83.9% (79.1%-87.7%)	89.0% (88.4%-89.6%)	15.9% (14.1%-17.9%)	99.6% (99.4%-99.7%)	42.17 (30.48-58.33)
Male Ages 60-69	623/12496 (5.0%)	0.949 (0.942-0.956)	91.3% (88.9%-93.3%)	87.0% (86.4%-87.6%)	27.0% (25.1%-28.9%)	99.5% (99.3%-99.6%)	70.60 (53.13-93.80)
Female Ages 70-79	430/10180 (4.2%)	0.908 (0.896-0.919)	85.6% (81.9%-88.6%)	80.9% (80.1%-81.7%)	16.5% (15.0%-18.1%)	99.2% (99.0%-99.4%)	25.14 (19.12-33.06)
Male Ages 70-79	764/10920 (7.0%)	0.926 (0.918-0.934)	91.4% (89.2%-93.2%)	78.9% (78.1%-79.7%)	24.5% (23.0%-26.2%)	99.2% (99.0%-99.4%)	39.47 (30.53-51.04)
Female Age>=80	471/6947 (6.8%)	0.870 (0.855-0.884)	89.6% (86.5%-92.0%)	65.3% (64.2%-66.5%)	15.8% (14.5%-17.3%)	98.9% (98.5%-99.1%)	16.22 (12.01-21.90)
Male Age>=80	562/5836 (9.6%)	0.886 (0.873-0.898)	93.8% (91.5%-95.5%)	65.3% (64.0%-66.6%)	22.4% (20.7%-24.1%)	99.0% (98.6%-99.3%)	28.36 (20.05-40.12)
Negative for Chronic Pulmonary Disease	3006/83551 (3.6%)	0.947 (0.944-0.950)	89.2% (88.1%-90.3%)	87.5% (87.2%-87.7%)	21.0% (20.3%-21.7%)	99.5% (99.5%-99.6%)	57.83 (51.44-65.02)
Positive for Chronic Pulmonary Disease	1051/14962 (7.0%)	0.915 (0.909-0.922)	91.5% (89.7%-93.1%)	77.2% (76.5%-77.9%)	23.3% (22.0%-24.6%)	99.2% (99.0%-99.3%)	36.55 (29.31-45.58)
Negative for Congestive Heart Failure	1424/81731 (1.7%)	0.939 (0.934-0.944)	81.5% (79.4%-83.5%)	90.8% (90.6%-91.0%)	13.6% (12.9%-14.3%)	99.6% (99.6%-99.7%)	43.52 (37.99-49.86)
Positive for Congestive Heart Failure	2633/16782 (15.7%)	0.866 (0.860-0.873)	94.3% (93.4%-95.1%)	58.5% (57.7%-59.4%)	29.7% (28.8%-30.7%)	98.2% (97.9%-98.5%)	23.38 (19.76-27.66)
Negative for Diabetes	2862/83337 (3.4%)	0.947 (0.944-0.951)	88.6% (87.4%-89.7%)	88.2% (88.0%-88.4%)	21.1% (20.4%-21.8%)	99.5% (99.5%-99.6%)	58.18 (51.74-65.42)
Positive for Diabetes	1206/15426 (7.8%)	0.907 (0.901-0.914)	92.7% (91.1%-94.0%)	73.2% (72.5%-74.0%)	22.7% (21.6%-23.9%)	99.2% (99.0%-99.3%)	34.76 (27.89-43.32)
Negative for Hypertension, combined	1679/53693 (3.1%)	0.960 (0.956-0.964)	89.4% (87.8%-90.8%)	90.9% (90.6%-91.1%)	24.0% (22.9%-25.1%)	99.6% (99.6%-99.7%)	83.75 (71.50-98.11)
Positive for Hypertension, combined	2378/44820 (5.3%)	0.921 (0.916-0.926)	90.1% (88.9%-91.3%)	80.0% (79.6%-80.3%)	20.1% (19.4%-20.9%)	99.3% (99.2%-99.4%)	36.40 (31.75-41.74)
Negative for Myocardial Infarction	3352/90058 (3.7%)	0.948 (0.945-0.951)	89.7% (88.6%-90.7%)	87.3% (87.0%-87.5%)	21.4% (20.7%-22.1%)	99.5% (99.5%-99.6%)	59.58 (53.21-66.71)
Positive for Myocardial Infarction	705/8455 (8.3%)	0.886 (0.876-0.897)	90.5% (88.1%-92.4%)	71.3% (70.3%-72.3%)	22.3% (20.8%-23.8%)	98.8% (98.5%-99.1%)	23.65 (18.30-30.56)
Negative for Obesity	2645/66174 (4.0%)	0.944 (0.940-0.947)	89.8% (88.5%-90.9%)	86.2% (86.0%-86.5%)	21.4% (20.6%-22.1%)	99.5% (99.4%-99.6%)	54.89 (48.31-62.37)
Positive for Obesity	1423/32579 (4.4%)	0.941 (0.936-0.946)	90.0% (88.3%-91.4%)	85.4% (85.0%-85.8%)	21.9% (20.9%-23.0%)	99.5% (99.4%-99.5%)	52.29 (43.86-62.33)
Negative for Renal Disease	2651/84400 (3.1%)	0.949 (0.945-0.952)	87.9% (86.6%-89.1%)	88.7% (88.5%-89.0%)	20.2% (19.5%-21.0%)	99.6% (99.5%-99.6%)	57.25 (50.85-64.47)
Positive for Renal Disease	1406/14113 (10.0%)	0.891 (0.884-0.899)	93.5% (92.0%-94.6%)	68.0% (67.2%-68.8%)	24.4% (23.3%-25.6%)	98.9% (98.7%-99.1%)	30.40 (24.53-37.67)
Overall	4068/98763 (4.1%)	0.943 (0.940-0.945)	89.8% (88.9%-90.7%)	86.0% (85.7%-86.2%)	21.6% (20.9%-22.2%)	99.5% (99.4%-99.5%)	54.03 (48.73-59.91)

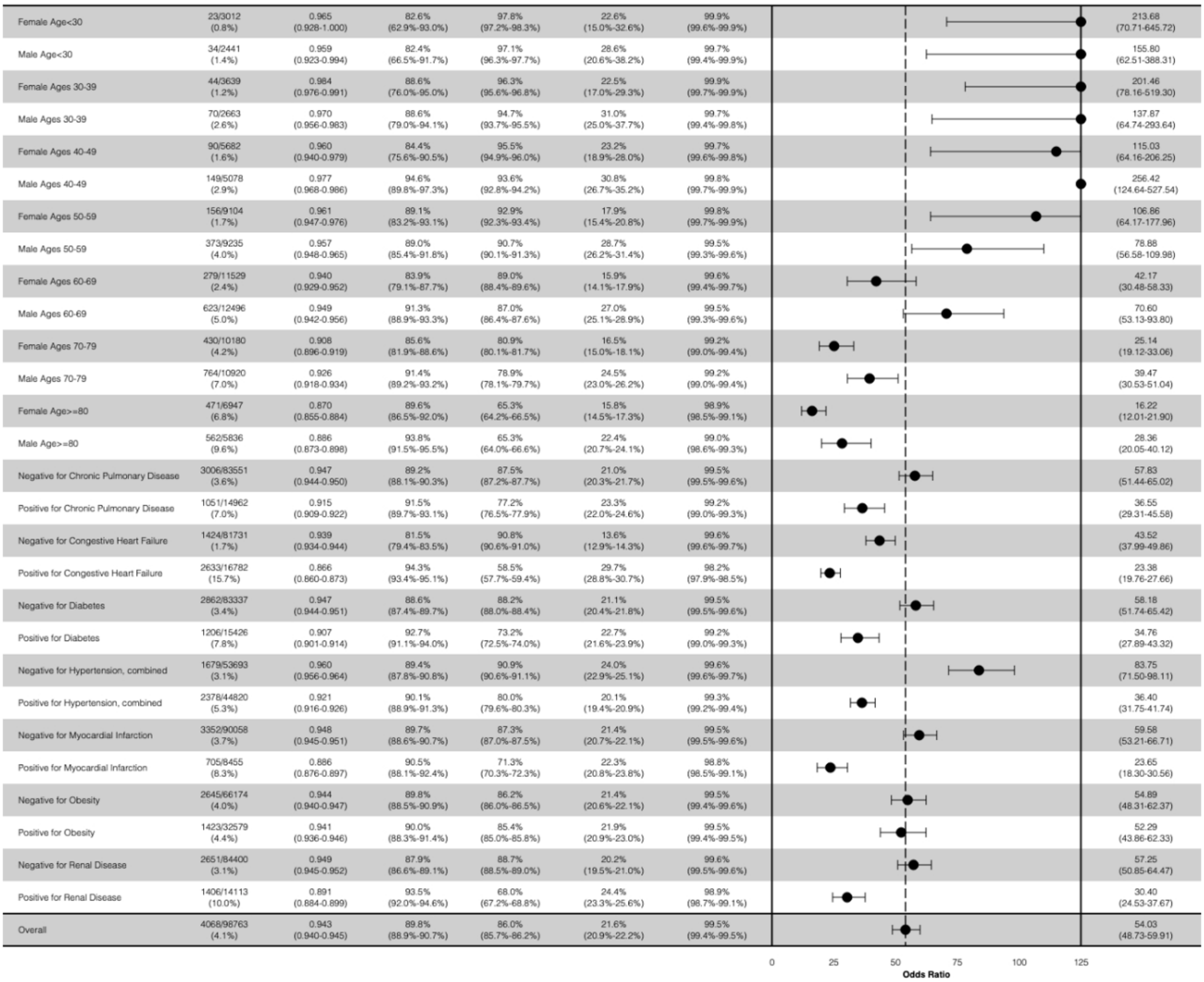

Figure 2. The distribution of left atrial volume index, e', E/e', tricuspid regurgitation velocity, and E/A according to filling pressure by our AI-ECG and echocardiography in test set. Each point represents the mean value with 95% confidence interval. Normal filling pressure includes normal and grade1, and increased filling pressure includes grade 2 and grade 3.

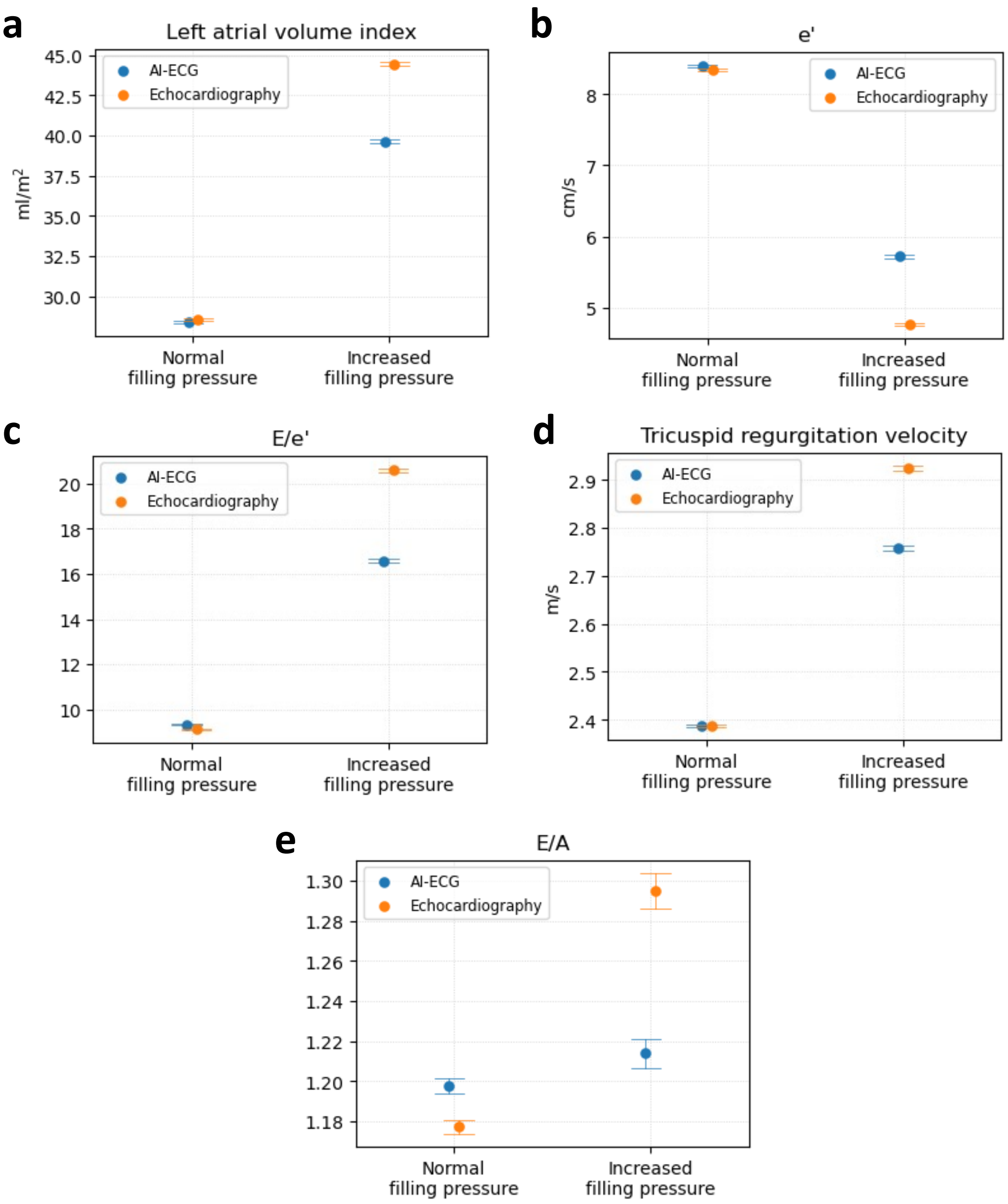

Figure 3. The distribution of left atrial volume index, e', E/e', tricuspid regurgitation velocity, and E/A according to filling pressure by our AI-ECG in indeterminate set. Each point represents the mean value with 95% confidence interval. Normal filling pressure includes normal and grade1, and increased filling pressure includes grade 2 and grade 3. Student's t-test is used for *p*-value.

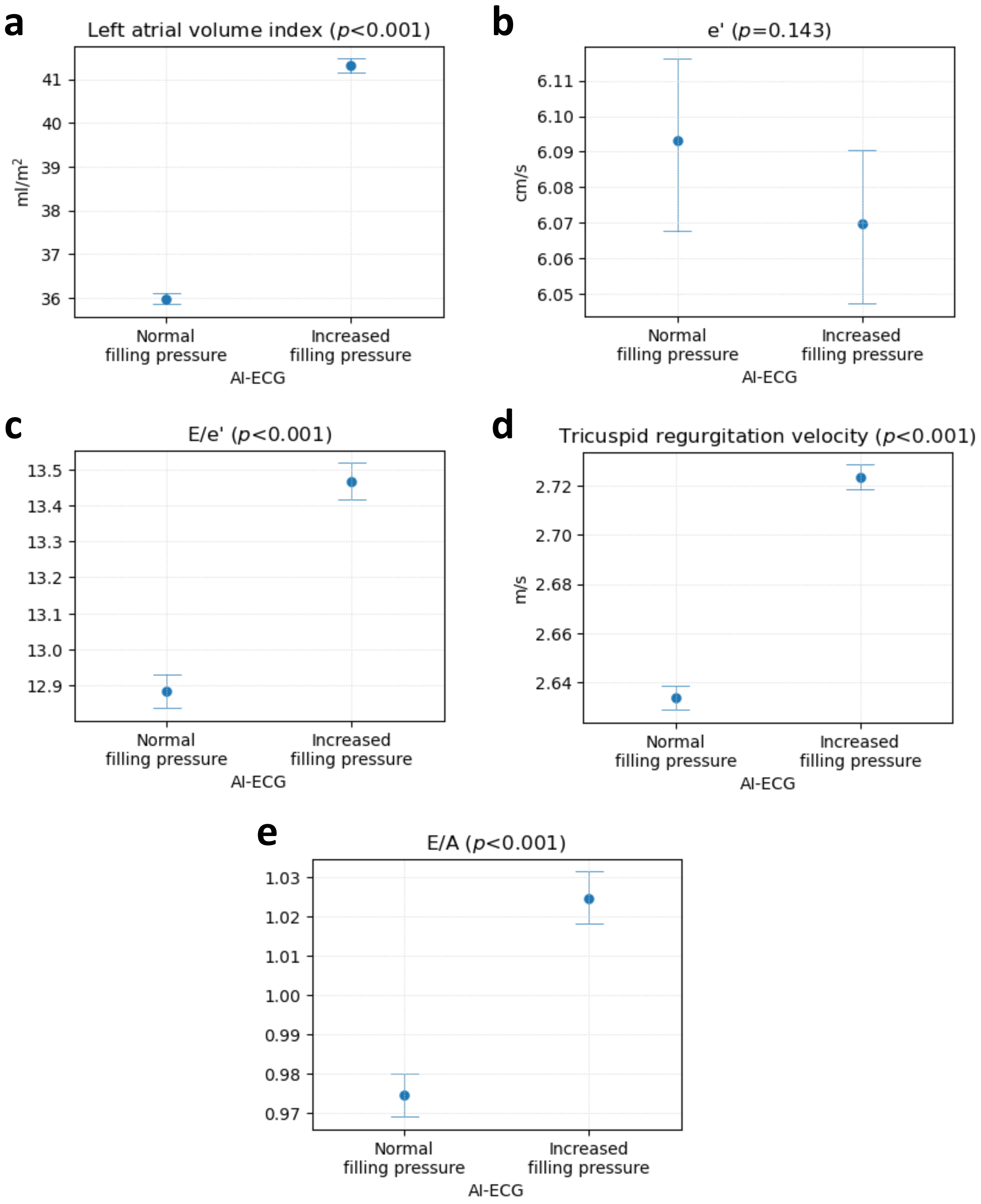

Figure 4. The ROC curves of AI-ECG between before and after the median year of the echocardiography exam in the test set. ROC plots for detecting diastolic function grades using ordinal scale.

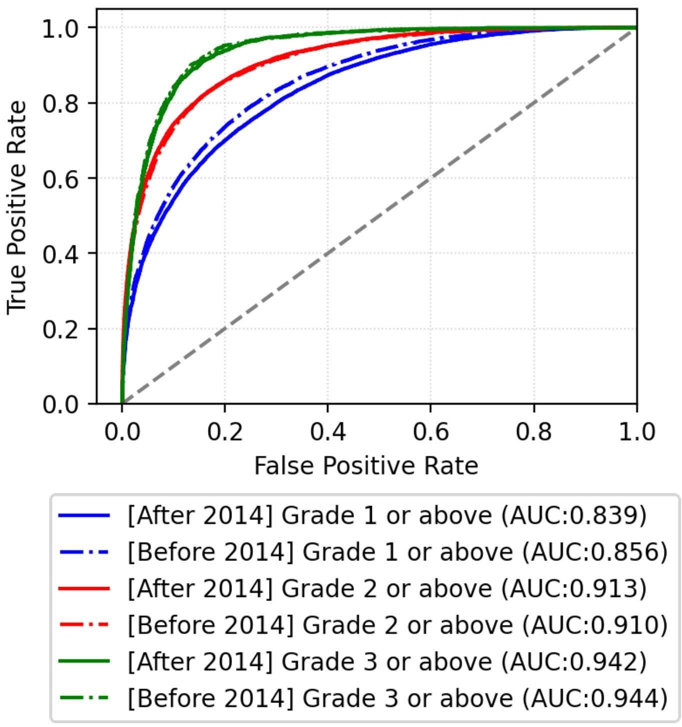

Figure 5. All-cause mortality using a Kaplan-Meier curve with 95% confidence intervals and p-value from a log-rank test by true positive (TP), false positive (FP), false negative (FN), and true negative (TN) for grade 1 or above, grade 2 or above, and grade 3.

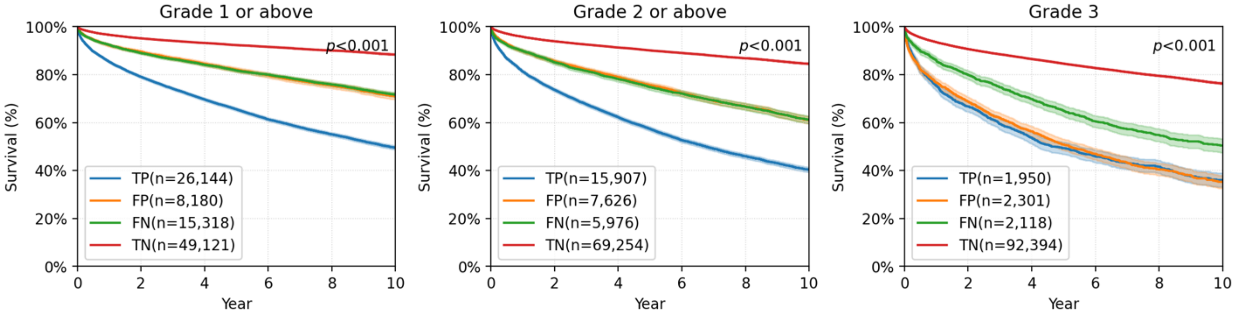

Figure 6. All-cause mortality using a Kaplan-Meier curve with 95% confidence intervals and p-value from a log-rank test by age. a. Kaplan-Meier curve for test group of patients (age≤50) according to the filling pressure by our AI-ECG (HR 1.413, 95% CI 1.329-1.503). b. Kaplan-Meier curve for test group of patients (50< age <70) according to the filling pressure by our AI-ECG (HR 1.35, 95% CI 1.313-1.388). c. Kaplan-Meier curve for test group of patients (age≥70) according to the filling pressure by our AI-ECG (HR 1.235, 95% CI 1.211-1.26). HRs were calculated with four grades after adjusting by age, sex, and comorbidities.

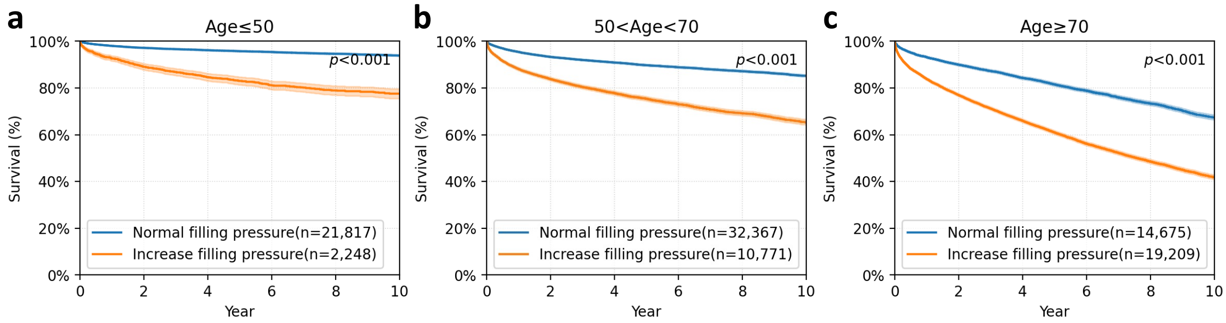

Figure 7. The distribution of left atrial volume index, e', E/e', tricuspid regurgitation velocity, and E/A according to diastolic filling pressure by our AI-ECG in grade 1 by echocardiography. Each point represents the mean value with 95% confidence interval. Normal filling pressure includes normal and grade1, and increased filling pressure includes grade 2 and grade 3. Student's t-test is used for *p*-value.

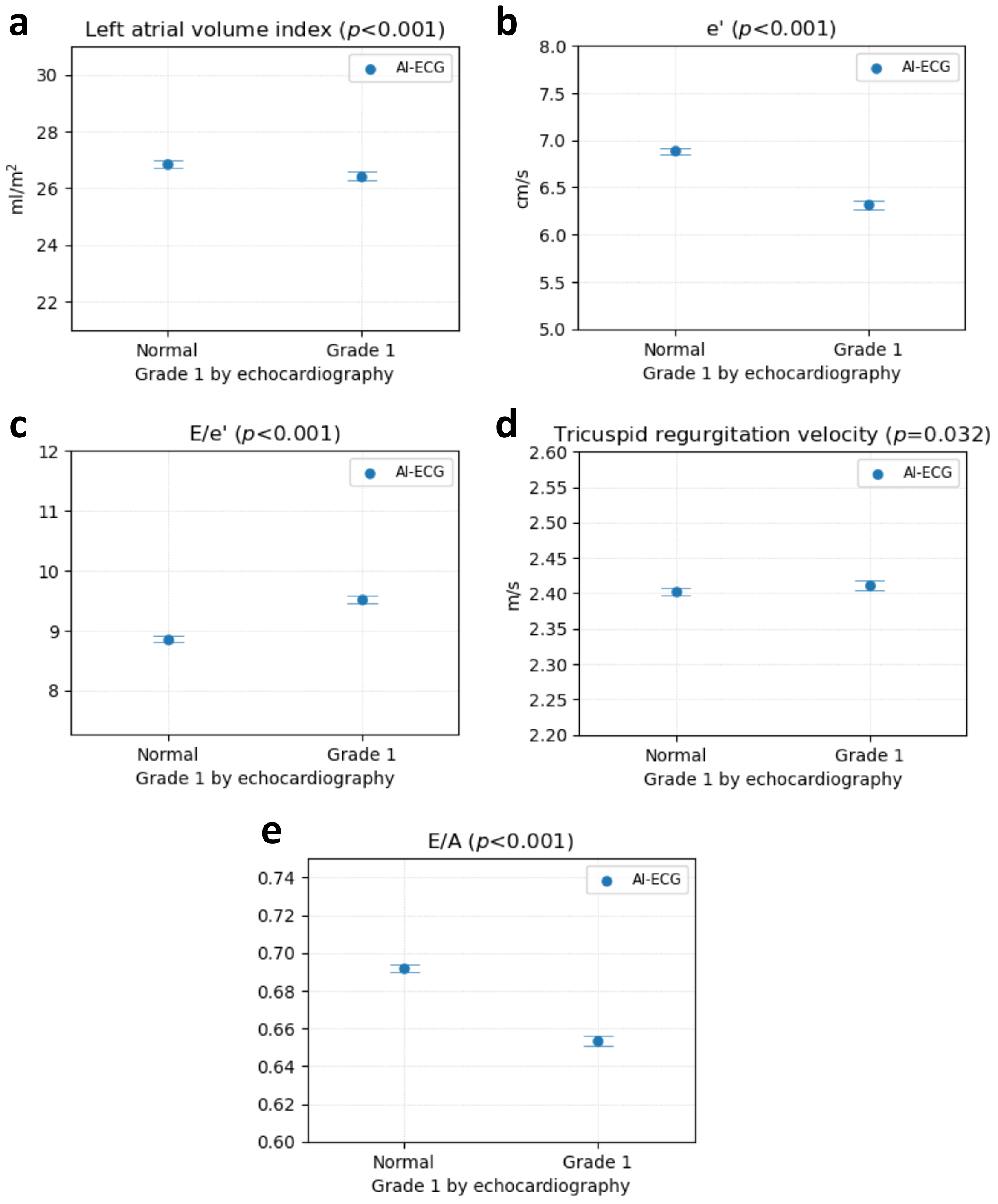

Figure 8. The ROC curves and performance of AI-ECG in normal ejection fraction and low ejection fraction, defined as left ventricular ejection fraction <50% in the test set. ROC plots for detecting diastolic function grades using ordinal scale.

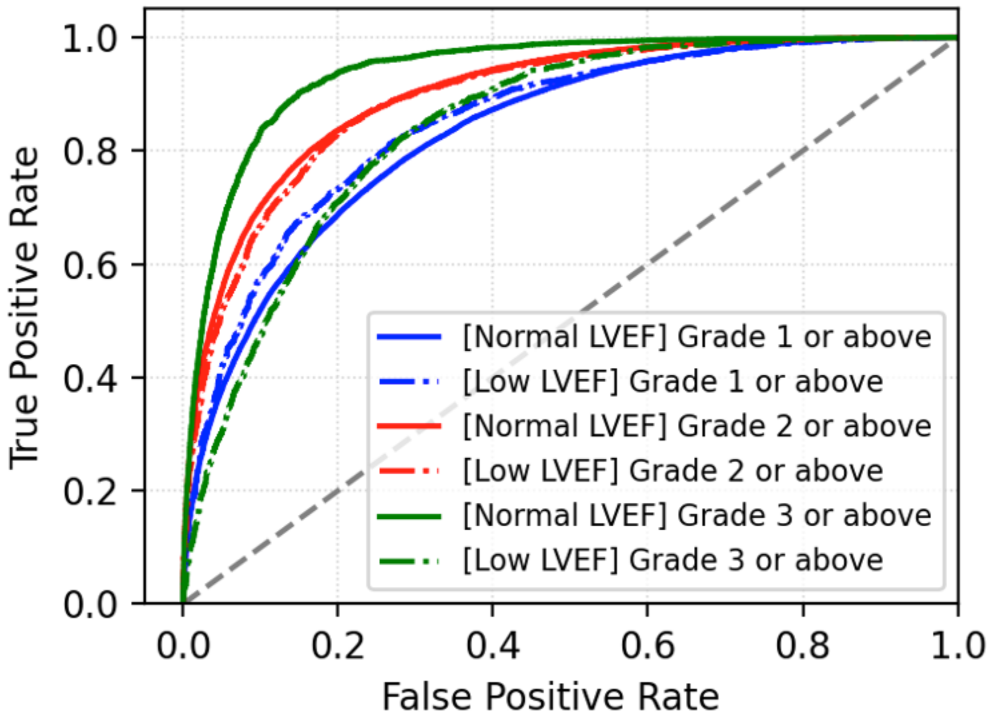

Ordinal grade	LVEF	AUROC	Sensitivity	Specificity	PPV	NPV
Grade 1 or above	Normal	0.83	0.58	0.87	0.73	0.77
	Low	0.85	0.87	0.64	0.89	0.58
Grade 2 or above (Increased filling pressure)	Normal	0.90	0.67	0.91	0.63	0.92
	Low	0.89	0.90	0.69	0.82	0.82
Grade 3 or above	Normal	0.94	0.36	0.99	0.40	0.98
	Low	0.84	0.62	0.85	0.51	0.90

Figure 9. An illustrative case of a heart failure patient. Video for the echocardiograms is attached separately (Supplemental Video 1).

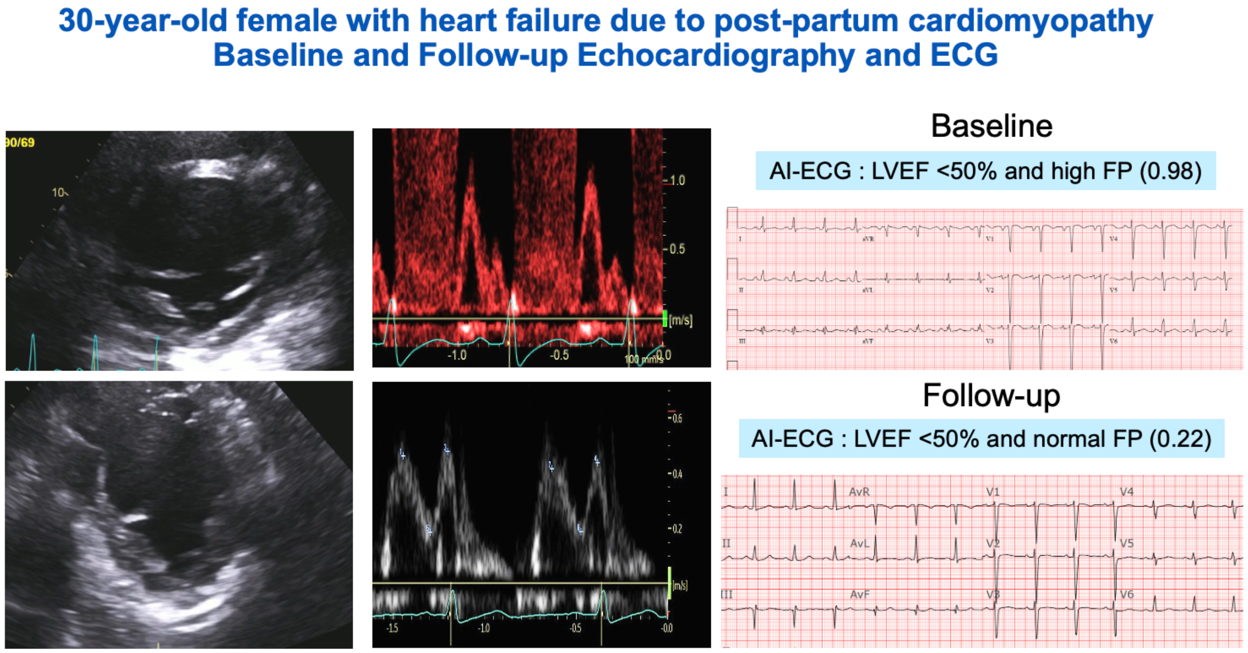

Figure 10. Flow chart of dataset construction and patient distribution. a. A flow chart demonstrating patient selection. b. A pie chart for dataset split.

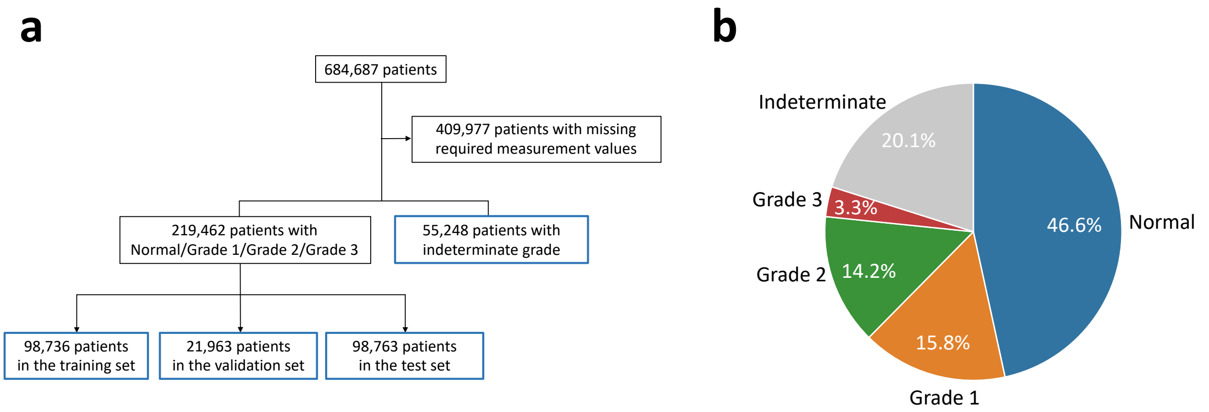

Figure 11. Algorithm for assessment of diastolic filling pressure and function[1].

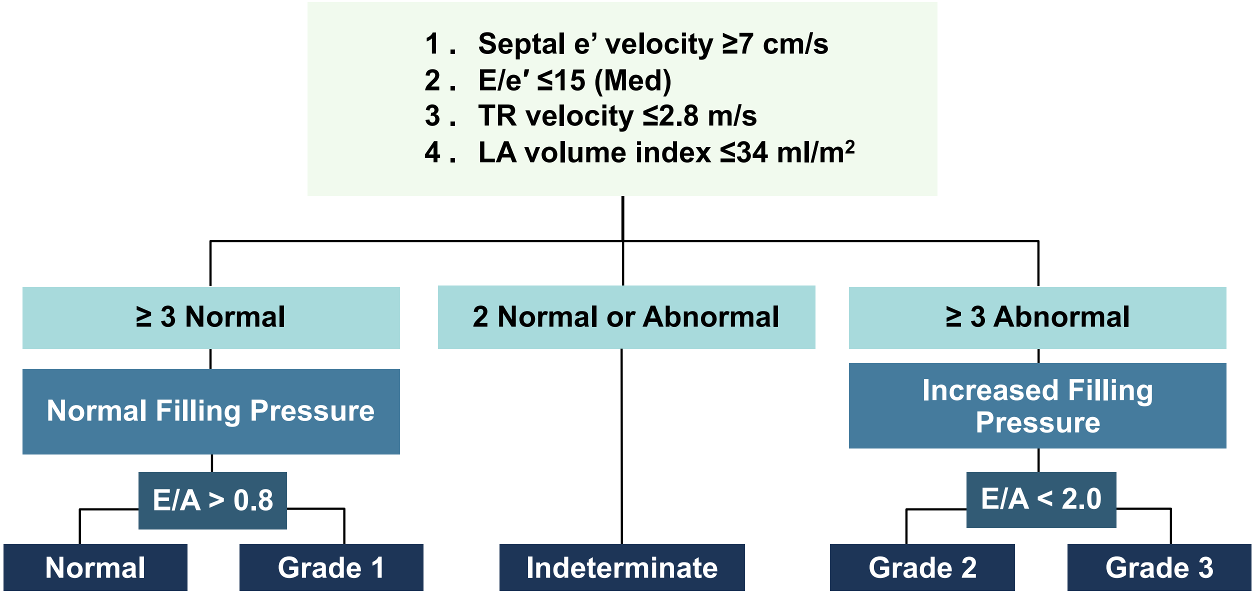

A=Late mitral inflow velocity with atrial contraction, E=Early diastolic mitral inflow velocity, e'=early medial (Med) or septal mitral annuls diastolic velocity, LA=Left atrium, TR=Tricuspid regurgitation velocity.

[1] Oh JK, Miranda WR, Bird JG, Kane GC, Nagueh SF. The 2016 diastolic function guideline: is it already time to revisit or revise them? JACC: Cardiovascular Imaging 2020;13(1 Part 2):327-335.

2. **Table**

Table 1. **Patient characteristics of training, validation, and testing group.** Values are n (%) or mean ± SD. Obesity was defined as body mass index ≥30. Renal disease includes any stages of chronic kidney disease, hypertensive kidney disease, glomerulonephritis, nephritic syndrome, hereditary nephropathy, end stage renal disease, unspecified kidney failure, dialysis, and kidney transplant status.

	**Overall (n=219,462)**		
	**Training (n=98,736)**	**Validation (n=21,963)**	**Test (n=98,763)**
**Age, y**	61.0 ± 16.8	60.8 ± 16.8	61.2 ± 16.8
**Female sex, Number (%)**	50,574 (51.2%)	11,164 (50.8%)	50,093 (50.7%)
**Myocardial infarction, Number (%)**	8,513 (8.6%)	1,914 (8.7%)	8,455 (8.6%)
**Congestive heart failure, Number (%)**	16,797 (17.1%)	3,833 (17.5%)	16,782 (17.0%)
**Cerebrovascular disease, Number (%)**	9,082 (9.2%)	1,970 (9.0%)	9,177 (9.3%)
**Chronic pulmonary disease, Number (%)**	14,869 (15.1%)	3,277 (15.0%)	14,962 (15.2%)
**Diabetes Mellitus, Number (%)**	15,598 (15.8%)	3,504 (16.0%)	15,426 (15.6%)
**Renal disease, Number (%)**	13,825 (14.0%)	3,086 (14.1%)	14,113 (14.3%)
**Hypertension, Number (%)**	44,639 (45.3%)	9,888 (45.1%)	44,820 (45.5%)
**Obesity, Number (%)**	32,696 (33.1%)	7,280 (33.1%)	32,579 (33.0%)

Table 2. Patient characteristics of four diastolic grade groups determined by AI-ECG in test set. Values are n (%).

	Test (n=98,763)			
**AI-ECG diastolic function grade**	**Normal (n=64,439)**	**Grade 1 (n=10,791)**	**Grade 2 (n=19,282)**	**Grade 3 (n=4,251)**
**Age, year**	55.6 ± 16.1	70.0 ± 10.5	73.0 ± 12.7	69.8 ± 14.7
**Female sex, Number (%)**	32,801 (50.9%)	5,338 (49.5%)	10,340 (53.6%)	1,614 (38.0%)
**Myocardial infarction, Number (%)**	3,674 (5.7%)	1,071 (9.9%)	2,897 (15.1%)	813 (19.2%)
**Congestive heart failure, Number (%)**	5,425 (8.4%)	1,414 (13.1%)	7,012 (36.5%)	2,931 (69.1%)
**Cerebrovascular disease, Number (%)**	4,513 (7.0%)	1,341 (12.4%)	2,793 (14.5%)	530 (12.5%)
**Chronic pulmonary disease, Number (%)**	7,848 (12.2%)	1,760 (16.3%)	4,164 (21.7%)	1,190 (28.1%)
**Diabetes, Number (%)**	6,961 (10.8%)	1,901 (17.6%)	5,188 (26.9%)	1,376 (32.4%)
**Renal disease, Number (%)**	5,694 (8.9%)	1,427 (13.2%)	5,329 (27.7%)	1,663 (39.2%)
**Hypertension, Number (%)**	23,310 (36.3%)	6,151 (57.1%)	12,825 (66.7%)	2,534 (59.7%)
**Obesity, Number (%)**	20,356 (31.6%)	4,060 (37.6%)	6,847 (35.5%)	1,316 (31.0%)

Table 3. Model performance for filling pressure and diastolic function grade from the AI-ECG in test set by hypertension, obesity, and diabetes. The threshold from the entire validation were used for each class.

Comorbidity	Class	Prevalence	AUCROC	Sensitivity	Specificity	PPV	NPV
**Hypertension**	**Grade 1 or above**	Y	0.81	0.8	0.65	0.72	0.74
		N	0.86	0.67	0.86	0.6	0.89
	**Grade 2 or above**	Y	0.88	0.84	0.76	0.6	0.92
		N	0.94	0.8	0.91	0.5	0.98
	**Grade 3**	Y	0.93	0.9	0.81	0.21	0.99
		N	0.97	0.9	0.93	0.25	1
**Obesity**	**Grade 1 or above**	Y	0.82	0.77	0.7	0.67	0.79
		N	0.86	0.76	0.78	0.7	0.83
	**Grade 2 or above**	Y	0.9	0.82	0.81	0.58	0.93
		N	0.92	0.84	0.84	0.58	0.95
	**Grade 3**	Y	0.94	0.9	0.85	0.22	0.99
		N	0.94	0.9	0.86	0.21	1
**Diabetes**	**Grade 1 or above**	Y	0.81	0.83	0.61	0.75	0.71
		N	0.85	0.74	0.78	0.66	0.84
	**Grade 2 or above**	Y	0.89	0.86	0.73	0.65	0.9
		N	0.91	0.81	0.85	0.54	0.95
	**Grade 3**	Y	0.91	0.92	0.76	0.22	0.99
		N	0.95	0.88	0.89	0.21	1

Table 4. Clinical characteristics of normal or grade 1 group by AI-ECG in grade 1 patients by echocardiography. Values are n (%) or mean ± SD. Student t-test and chi-squared test were used.

	**Normal (n=10,712)**	**Grade 1 (n=5,669)**	***p*-value**
**Age, y**	66.1 ± 10.9	71.6 ± 9.1	<0.001
**Female sex, Number (%)**	5,194 (48.5%)	2,823 (49.8%)	0.861
**Myocardial infarction, Number (%)**	799 (7.5%)	512 (9.0%)	<0.001
**Congestive heart failure, Number (%)**	1,063 (10.0%)	747 (13.2%)	<0.001
**Cerebrovascular disease, Number (%)**	1,064 (10.0%)	737 (13.0%)	<0.001
**Chronic pulmonary disease, Number (%)**	1,608 (15.1%)	935 (16.5%)	0.015
**Diabetes Mellitus, Number (%)**	1,643 (15.3%)	1,001 (17.7%)	<0.001
**Renal disease, Number (%)**	1,185 (11.1%)	788 (13.9%)	<0.001
**Hypertension, Number (%)**	5,319 (49.8%)	3,350 (59.1%)	<0.001
**Obesity, Number (%)**	3,565 (33.3%)	2,044 (36.1%)	<0.001

Table 5. The number of patients for hypertrophic cardiomyopathy (HCM), cardiac amyloidosis, moderate to severe mitral valve regurgitation (MR) and aortic stenosis (AS), and reduced left ventricular ejection fraction (LVEF) according to diastolic function grade by AI-ECG. Percent in each column indicates distribution of a particular condition within the same diastolic function category.

	**Test (n=98,763)**			
**AI-ECG diastolic function grade**	**Normal (n=64,439)**	**Grade 1 (n=10,791)**	**Grade 2 (n=19,282)**	**Grade 3 (n=4,251)**
**HCM**	1,103 (1.7%)	233 (2.2%)	1,267 (6.6%)	852 (20%)
**Amyloidosis**	2,590 (4%)	373 (3.5%)	1,571 (8.1%)	2,225 (52.3%)
**Moderate to severe MR**	1,070 (1.7%)	144 (1.3%)	1,958 (10.2%)	1,017 (23.9%)
**Moderate to severe AS**	588 (<1%)	242 (2.2%)	1,374 (7.1%)	265 (6.2%)
**Reduced LVEF (<50%)**	2,252 (3.5%)	755 (7%)	3,813 (19.8%)	2,252 (53%)
